# An Azobenzene‐Based Liquid Molecular Solar Thermal (MOST) Storage System–Energy Carrier and Solvent

**DOI:** 10.1002/smll.202502938

**Published:** 2025-06-02

**Authors:** Dominic Schatz, Conrad Averdunk, Rouven Fritzius, Hermann A. Wegner

**Affiliations:** ^1^ Institute of Organic Chemistry Justus Liebig University Giessen Heinrich‐Buff‐Ring 17 35392 Gießen Germany; ^2^ Center of Materials Research (ZfM/LaMa) Justus Liebig University Giessen Heinrich‐Buff‐Ring 16 35391 Giessen Germany

**Keywords:** azo compounds, electrochemistry, energy storage, photochemistry, solvent

## Abstract

A molecular solar thermal (MOST) storage systems is based on capturing solar energy via photoisomerization, which can be released later as thermal energy. Herein, the low viscosity, green light active, 2,6‐difluoroazobenzene is introduced, which can be efficiently irradiated, pumped, and handled in its neat state. Synthesis as well as isomerization can be done conveniently in a continuous flow setup. Storage densities of 218 kJ kg^−1^ for 100% (*Z*)‐isomer (137 kJ kg^−1^ after green light irradiation) are the highest compared to other liquid azobenzenes (ABs). Additionally, the irradiation with green light and the processibility in the neat state make this compound a promising candidate for energy storage applications. Furthermore, the liquid AB can be employed as a MOST‐active solvent. For example, the solvation of an electrolyte is demonstrated to induce a measurable conductivity, which then allows for complete electron‐catalyzed back‐isomerization. Alternatively, it can act as a solvent for a higher energy MOST material. As a proof‐of‐concept a norbornadiene (NBD) is dissolved in the AB solvent allowing to utilize the energy of the NBD as well as the AB solvent. Further optimization of the solute‐solvents systems is required to fully harvest the potential of this new concept for efficient energy storage.

## Graphical abstract

A low viscous *ortho*‐fluorinated azobenzene is introduced as a solvent‐free energy storage material. It can be prepared, charged, and discharged in a continuous‐flow setup. As it is a low molecular weight liquid molecule, it can be used to dissolve higher energy density norbornadienes to act as a MOST‐active solvent and dissolve an electrolyte to increase its conductivity for electrochemical‐triggered heat release.

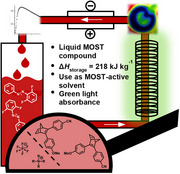

## 1 Introduction

**Figure 1:**
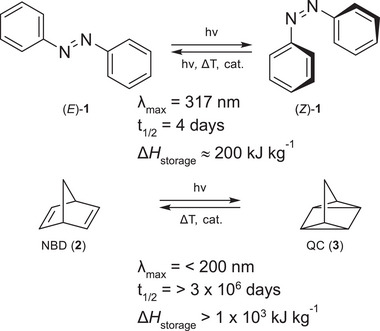
Selected molecular photoswitches utilized in the context of MOST systems.^[^ ^6^, ^9^ ^]^ AB **1** can be isomerized from stable (*E*)‐ to metastable (*Z*)‐isomer, and NBD (**2**) can be transformed to the QC (**3**) structure.

As the worldwide energy demand grows, and the negative effect of fossil fuel consumption becomes prevalent, renewable energy resources become an important topic in research, as well as in politics and society. The ever‐increasing demand for sustainable energy production,^[^ ^1^, ^2^ ^]^ storage, and conversion lead to a variety of promising technologies, like photovoltaic devices,^[^ ^3^ ^]^ redox‐flow batteries,^[^ ^4^ ^]^ or efficient water splitting reactions.^[^ ^5^ ^]^ Another enabling concept that utilizes the source of the most abundant renewable energy – the sun – is molecular solar thermal storage (MOST) systems.^[^ ^6^, ^7^, ^8^ ^]^ The ideas behind this technology rely on the property of molecular photoswitches which can be converted from a thermodynamic stable ground state to a higher‐energy metastable state by irradiation with a specific wavelength. Back‐isomerization to the ground state then releases the stored thermal energy. There are multiple scaffolds that can be applied as energy storage systems, each with its own advantages and disadvantages. Azobenzenes [AB, from (*E*)‐ to (*Z*)‐isomerization] and norbornadiene [from norbornadiene (NBD) to quadricyclane (QC)] are the most explored examples (**Figure** **1**).

Important parameters that need to be addressed and optimized for MOST systems are the solar spectrum match, storage time, energy density, energy release, quantum yields, and stability. In the case of AB, the higher energy (*Z*)‐state can be reached by exciting the *π*–*π* ^*^ transition by irradiation at the absorption maxima. To achieve isomerization with visible light and increase the solar match, red‐shifting of the *π*–*π* ^*^ band is needed. Due to the prevalent use of AB in photopharmacology, these properties have been studied in depth.^[^ ^10^, ^11^, ^12^ ^]^ Introduction of a push‐pull system across the diazo unit can shift the absorption maxima into the red region, but usually results in a very fast thermal relaxation time.^[^ ^13^ ^]^ As a large half‐life is needed for most storage applications of the absorbed energy, a different way to induce green light absorption is required. An additional possibility for photoisomerization is irradiation into the n–π^*^ band. Usually, the n–π^*^ bands of (*E*)‐ and (*Z*)‐AB are superimposed, so that the resulting photostationary state (PSS) does not result in adequate (*Z*)‐isomer formation. This overlap can be decreased by *ortho* substitution. Wooley introduced methoxy groups in all four *ortho* positions and observed a shift in the n–π^*^ maxima of both isomers by 28 nm. This is due to the repulsion of the methoxy groups and the nitrogen lone pairs, which is more pronounced in the (*E*)‐state, thus red‐shifting the n–π^*^ band of the (*E*)‐isomer, while the (*Z*)‐isomer is less effected. Hecht and Bléger pioneered the *ortho*‐substitution with electron‐withdrawing fluorine substituents, which stabilize the n‐orbital of the (*Z*)‐isomer, as well as the π^*^‐orbitals of both isomers, resulting in efficient net separation of the absorption of both isomers. An additional effect of the stabilized (*Z*)‐n‐orbital is a long half‐life, which is important for MOST materials. The effect of an electron‐rich or electron‐poor substituent in *ortho*‐position for red shifting has been exploited by the Li group for 1,3‐bis‐hetero AB,^[^ ^14^ ^]^ and for mono‐hetero AB,^[^ ^15^ ^]^ by the Han and Feng group for phase‐change and liquid storage materials.^[^ ^16^, ^17^ ^]^

Next to the isomerization wavelength, a large energy density, low molecular‐weight compounds that show good solubility are important for efficient energy storage. If the application of interest allows solid‐state photoswitches to be used, the photoisomerization can be accompanied by a phase transition.^[^ ^18^ ^]^ The liquefication upon irradiation stores additional energy in the form of the phase transition, while the crystallization upon back isomerization releases isomerization energy as well as lateral heat. Another way to increase the energy density is to stabilize the (*E*)‐isomer, e.g. through intermolecular attractive interactions,^[^ ^19^ ^]^ such as London dispersion.^[^ ^20^ ^]^ Additionally, multiple AB units can be fused to a single aromatic ring, therefore lowering the molecular mass per switching unit.^[^ ^21^ ^]^

**Figure 2:**
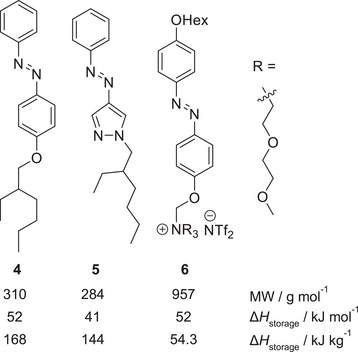
Properties of liquid state AB compounds **4**–**6** reported by Kimizuka in the literature.^[^ ^22^, ^29^, ^30^, ^31^ ^]^ Energy densities are calculated for 100% (*Z*)‐isomer.

One of the reasons that prevents MOST molecules from achieving their full potential is their application in solution. This introduces often toxic and expensive solvents that, while allowing easy handling of the MOST compound, are lowering the energy density tremendously and diminishing the maximum obtainable thermal energy. Therefore, it would be important to avoid unnecessary solvents completely. The first example of such a compound was reported by Kimizuka in 2014, which is based on branched 2‐ethylhexoxy AB **4** (**Figure** **2**).^[^ ^22^ ^]^ In their seminal paper, they showed that the isomerization of AB can efficiently occur in the solvent‐free, condensed phase. Differential scanning calorimetry (DSC) measurements revealed, after irradiation with UV light, an energy density of 52 kJ mol^−1^, or 183 kJ L^−1^ [for presumed 100% (*E*)‐isomer]. In subsequent work from the Moth–Poulsen group the same AB **4** has been used in a device, which allows the *in fluxu* irradiation, as well as the *in fluxu* catalytic energy release with a copper(I) complex.^[^ ^23^ ^]^ Unfortunately, the liquid AB was again employed in µM concentration, probably to facilitate irradiation and transportation through a chip reactor. Additionally, most liquid AB rely on the introduction of long, sometimes branched, alkyl chains, by adding flexibility and to hinder *π–π* interactions of the phenyl units. Other methods rely on the introduction of asymmetry,^[^ ^24^, ^25^, ^26^ ^]^ or by inducing a twist around the azo moiety.^[^ ^27^ ^]^ These additional groups increase the synthetic effort, lower efficiency of the storage material, and decrease the energy density due to the additional molecular weight (Figure 2). There is tremendous effort to obtain higher energy densities, but most devices still require a solvent for efficient solar harvest and transport, thereby lowering the applicability. Additionally, the usage of toxic organic solvents is not in compliance with these greener, solar technologies.

In 2014, Hecht and Bléger introduced *ortho*‐fluoro ABs as a green light, high quantum yield, long half‐life photoswitchable molecules, and reported the non‐symmetric 2,6‐difluoro AB (**10**, *o*F‐AB, MW = 218 g mol^−1^) as an oil.^[^ ^28^ ^]^ Herein, we investigate this liquid AB as a visible light photoswitch in a solvent‐free solar thermal storage application. A focus here will be on the performance of the complete MOST cycle, from irradiation to heat release, without additional solvent. Moreover, we investigate the ability of the liquid AB to act as solvent to dissolve MOST compounds with higher energy density such as NBDs. With this new strategy, a better spectral overlap and a higher energy density could be achieved, while still having an easy to handle, low viscous liquid MOST material.

## 2 Results and Discussion

### 2.1 Synthesis

**Scheme 1:**
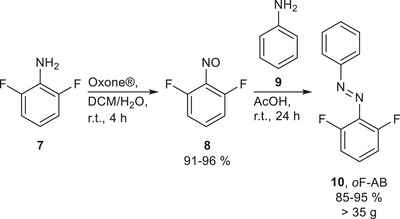
Biphasic oxidation of difluoro aniline **7** using Oxone to the nitrosobenzene **8**, followed by Baeyer–Mills reaction with aniline to yield the liquid AB **10** after distillation.

An efficient way to prepare non‐symmetric AB is the condensation of aniline and nitrosobenzene in a Baeyer–Mills reaction.^[^ ^32^, ^33^ ^]^ This reaction proceeds via a nucleophilic attack of the aniline on the nitrosobenzene under acidic or basic conditions,^[^ ^34^ ^]^ which explains why electron‐rich anilines and electron‐poor nitrosobenzenes provide high yields under mild reaction conditions and short reaction times. The *o*F‐AB **10** was prepared by oxidation of 2,6‐difluoroaniline (**7**) to the corresponding difluoro‐nitrosobenzene (**8**) with Oxone in a biphasic CH_2_Cl_2_/H_2_O mixture, which, after extraction and removal of the solvent, was employed in a Baeyer–Mills reaction in acetic acid (**Scheme** **1**).^[^ ^28^ ^]^

Instead of a column chromatography compound **10** was purified by fractional distillation resulting in a yield of 95%. The use of distillation as the main purification step allows easy up‐scaling for large‐scale preparation of *o*F‐AB **10**. We were able to prepare up to 38 g of *o*F‐AB **10** in a single batch, albeit using a higher concentration of starting material resulted in a slightly lower yield of 85%.

**Figure 3:**
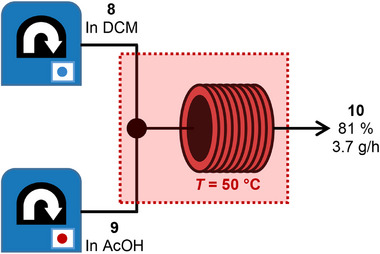
Schematic presentation of the continuous flow Baeyer–Mills reaction of aniline (**9**) and 2,6‐difluoronitrosobenzene (**8**) in a tubular reactor. A throughput of 3.7 g pure AB **10** was obtained after distillation.

A continuous flow setup offers advantages to batch synthesis, especially for large‐scale synthesis. We focused on the Baeyer–Mills reaction,^[^ ^35^ ^]^ as the oxidation from aniline to nitroso is limited by the solubility of Oxone. Nevertheless, it is also feasible to do both steps in flow by using a phase separator.^[^ ^36^ ^]^ As 2,6‐difluoro nitrosobenzene (**8**) is only slightly soluble in AcOH, we dissolved compound **8** in dichloromethane (DCM) and aniline (**9**) in AcOH. By increasing the reaction temperature to 50 °C, a flow‐rate of 2 mL min^−1^ (1 mL min^−1^ for compounds **7** and **8** respectively) can be achieved with a 10 mL coiled tube reactor (**Figure** **3**). Higher temperature results in the formation of phenazine derivative as a side product (Figure S22, Supporting Information).^[^ ^37^ ^]^ That way, continues flow yielded 24.8 g of clean *o*F‐AB **10** after distillation (runtime 400 min). This corresponds to a yield of 81%, and a throughput of purified compound of 3.7 g h^−1^. This yield is in accordance with the one we obtained by using higher concentrations in batch reactions. As flow synthesis is easy to up‐scale by either running the experiment longer, or by increasing the reactor volume, large‐scale synthesis of the AB can be assured.

### 2.2 Photophysical Properties

**Figure 4:**
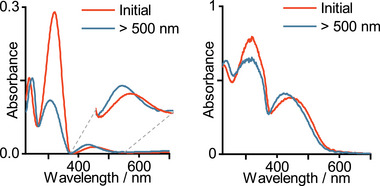
UV/Vis absorption spectra of *o*F‐AB **10** in ACN (left) solution and in the neat liquid state (right).

The UV/Vis absorption spectrum of *o*F‐AB **10** in acetonitrile (ACN) solution shows an absorption maximum of the π–π^*^ transition at 311 nm and a broad n–π^*^ transition centered at 450 nm (**Figure** **4**, left).

Upon irradiation with green light, the expected separation of the latter band is observed, accompanied by a decrease in absorption at 310 nm. A neat sample of *o*F‐AB **10** was prepared as a thin film between two quartz plates (Figure 4, right). The peak positions in the neat or dissolved state are very similar. The main difference is the much larger intensity of the formally forbidden n–π^*^ transition, which usually shows lower absorbance. This observation could be due to the difference in the transition dipole moments of both absorptions and a preferred alignment between the quartz plates.^[^ ^38^, ^39^ ^]^

Another important photophysical property for a potential MOST material is the thermal half‐life. The stability at room temperature needs to be high enough to ensure long‐time storability and minimize parasitic heat loss during storage. Hecht and Bléger obtained a thermal half‐life of 25 h of AB **10** at 60 °C in ACN solutions.^[^ ^28^ ^]^ As the measured half‐life is dependent on the environment,^[^ ^40^ ^]^ and a decrease of up to 87% of neat AB in comparison to a solution in ACN can be observed for hetero‐AB **5**,^[^ ^30^ ^]^ we measured the thermal stability without additional solvents. At 60 °C, we obtained a thermal half‐life of 22 h, which is very similar to the value obtained as a solution. Following an Eyring‐Polanyi analysis, we were able to estimate a half‐life at 25 °C of 53 days, which is well suited for MOST storage applications (Figure S5, Supporting Information). The high thermal stability allows *o*F‐AB **10** to be readily applied in large‐scale heat storage. To test the photochemical stability and to ensure longevity of our MOST material, cyclability studies with charging wavelengths of 530 nm and a higher energy alternative of 340 nm and discharging wavelength of 405 nm were performed (Figures S6 and S7, Supporting Information). We observed no notable degradation over > 30 cycles, and a maxima absorption retention if 100% and 99% were obtained for 530 and 340 nm respectively. This excellent photostability validates the practical applicability of photoswitch **10**.

### 2.3 Thermal Properties

The heat storage potential of *o*F‐AB **10** was investigated by DSC. A sample of neat *o*F‐AB  **10** was irradiated with a 530 nm LED, the content of the (*Z*)‐isomer was analyzed by HPLC, and measured from ‐100 °C to 200 °C. The obtained DSC thermogram shows a broad exothermic peak centered at ≈150 °C (Figure S8, Supporting Information), which is not present in the second heating/cooling procedure of the same sample (Figure S8, Supporting Information). We assigned this irreversible process to the thermal (*Z*)‐ to (*E*)‐isomerization of the *o*F‐AB **10**. At the PSS reachable with our green LED [PSS_530_ _nm_ = 63% (*Z*)‐isomer], a heat storage of 137 kJ kg^−1^ can be achieved, which corresponds to 218 kJ kg^−1^ for 100% (*Z*)‐isomer. This is considerably higher than the discussed liquid AB derivatives **4**–**6**, which again highlights advantage of the small molecular weight of our employed *o*F‐AB **10**. The group of Hecht reported a PSS of 74% with their green LED,^[^ ^28^ ^]^ which would correspond to a thermal energy storage of 161 kJ kg^−1^. The here obtained enthalpies are directly correlated to the maximum obtainable heat release, as no additional solvent is needed.

### 2.4 Irradiation

**Figure 5:**
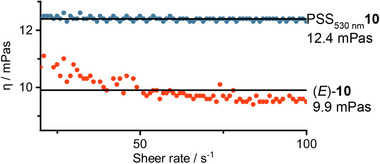
Rheology measurements of neat *o*F‐AB **10** in the ground state (*E*)‐isomer, and with 63% (*Z*)‐isomer at the PSS.

The irradiation of neat samples can be inefficient and tedious, in the solid state but also in the neat liquid state, mainly due to the inner filter effect and diffusion. We chose to rely on flow chemistry to tackle these difficulties. As the maximum flow rate and the back pressure of pumps depend on the viscosity of the employed liquid, it was important to quantify the viscosity of *o*F‐AB **10**. We measured the viscosity of (*E*)‐*o*F‐AB **10** and of the (*E*/*Z*)‐mixture at the PSS at 530 nm (**Figure** **5**). Interestingly, the *o*F‐AB **10** shows a larger shear viscosity at the PSS_530 nm_ then the (*E*)‐isomer. As the viscosity of liquids arises from friction between clustered molecules and is therefore dependent on the size, shape, and intermolecular interactions, there seems to be larger interactions within the metastable isomer. This is in contrast to the numerous examples of solid‐to‐liquid phase change AB, as well as to Kimizuka's liquid alkoxy AB **4**.^[^ ^22^ ^]^ Furthermore, the (*E*)‐isomer shows non‐Newtonian behavior in slow low shear rates. The obtained pseudoplastic flow is indicative of structural formations, which are disrupted by stress introduced with higher shear rates.^[^ ^41^ ^]^ The obtained viscosities are well within the range of transportable liquids, and a magnitude smaller than of an already used liquid energy AB^[^ ^22^, ^23^ ^]^ or liquid NBDs.^[^ ^42^ ^]^

**Figure 6:**
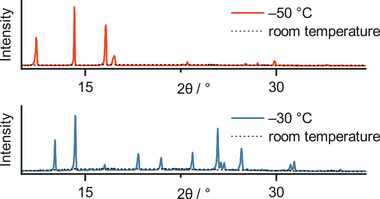
PXRD measurements of neat *o*F‐AB **10** in the ground state (*E*)‐isomer (top, orange trace), and with 63% (*Z*)‐isomer at the PSS_530 nm_ (bottom, blue trace). The room temperature measurement of both samples resulted in a broad background signal. Measurements were conducted in a thin capillary at room temperature and below the freezing temperature of both samples. Crystallinity was observed at −50 and −30 °C for (*E*) and PSS samples, respectively.

To further confirm the liquid state of the employed AB, temperature‐dependent powder X‐ray diffraction (PXRD, Figure S16, Supporting Information) was measured for the (*E*)‐isomer and the PSS_530 nm_ state (Figure S17, Supporting Information). For the (*E*)‐isomer, an unresolved broad background signal at room temperature confirms the appearance of an amorphous liquid state. This disordered diffraction behavior is present as well at −40 °C, but further cooling to −50 °C results in sharp peaks, indicating a crystalline pattern and therefore the freezing of the previously liquid sample (**Figure** **6**, top). For the PSS mixture, the broad amorphous signals is present at −20 °C, but already at −30 °C, crystallization is observed (Figure 6, bottom). Therefore, the (*E*)‐isomer shows a lower freezing point than the isomer mixture at 530 nm, but both mixtures are well within a standard operation temperature of a MOST material.

Clear freezing points cannot be observed during DSC experiments (Figure S8, Supporting Information). We propose that this can be explained by a slow crystallization kinetic, and the observable cold crystallization peak of the PSS sample could be an indication for this phenomena.^[^ ^43^ ^]^ The PXRD measurements were conducted in a thin capillary that offers enough surface area to induce crystallization, therefore allowing us to follow the phase change by their diffraction change.

**Figure 7:**
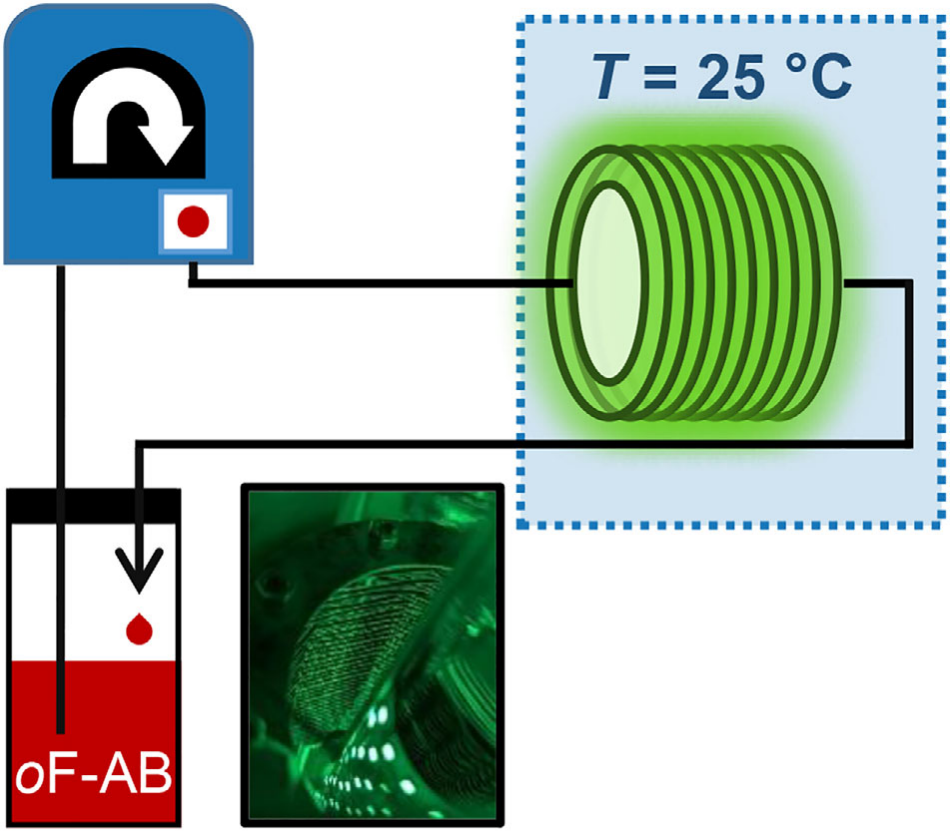
Continuous flow irradiation of n eat *o*F‐AB **10** in a 10 mL tube reactor.

Due to the low viscosity, and high fluidity nature of *o*F‐AB **10** over a wide temperature range, we can employ it in a photochemical flow setup for irradiation. Conducting photochemistry in flow has significant advantages compared to batch reactions. For example uniform irradiation, shorter reaction times, easy and efficient scale‐up, and less degradations due to better reaction control can be achieved.^[^ ^44^, ^45^, ^46^ ^]^ Especially the combination of flow chemistry with solar generators or collectors offers interesting possibilities for liquid MOST systems (**Figure** **7**).^[^ ^47^ ^]^

Depending on the residence time inside the irradiation chamber, different (*Z*)‐isomer ratios can be obtained. After ≈200 min, neat *o*F‐AB **10** was switched to the PSS of 63% (*Z*)‐isomer. Although the PSS is not ideal, it has been shown that a PSS up to 74% can be achieved by using a different green light source.^[^ ^28^ ^]^ Interestingly, we obtained even shorter irradiation times using a cheap commercially available LED strip, used for ambient light installations, and a simple home‐made weaved flow reactor (Figure 6). This highlights the easiness of real‐life implementations of this MOST system. With the chosen dimension of our reactor, this irradiation time would correspond to a throughput of 3.3 mL of charged *o*F‐AB **10** per hour. Due to the scalability of flow chemistry, increase of the reactor length and volume would linearly scale with our obtained throughput.

### 2.5 Energy Release

**Figure 8:**
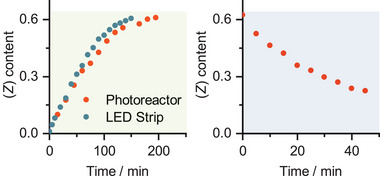
Change in the (*Z*)‐isomer content in the PSS during green light (left) and blue light (right) irradiation of neat *o*F‐AB **10** pumped through a 10 mL tube reactor. The irradiation was either conducted by a commercial photoreactor (530 and 405 nm), or by a simple home‐made setup using a LED strip intended for ambient light irradiation. During each run, 10 mL AB **10** was used to ensure a completely filled reactor. The (*Z*)‐content was measured via offline HPLC of aliquots integrated at the isosbestic point.

Irradiation with 405 nm leads to the back isomerization with a PSS of 78% of the (*E*)‐isomer (**Figure** **8**, right). As the PSS resulting from blue light irradiation is a limiting factor for the heat release, other triggers for the back reaction are necessary to harvest the maximum stored energy. One possibility is the electron‐catalyzed isomerization.^[^ ^48^ ^]^

**Figure 9:**
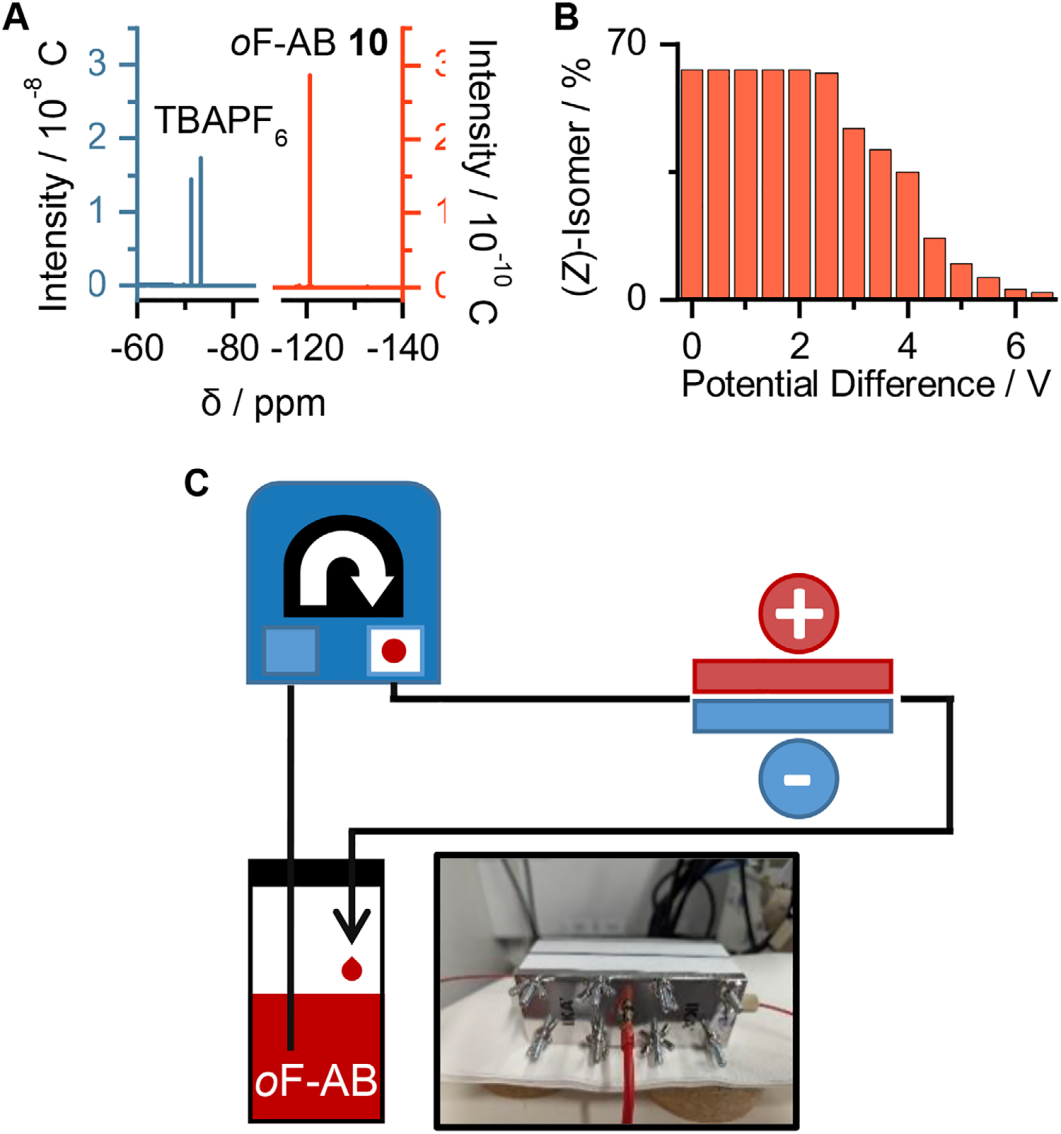
A) ^19^F‐NMR of a saturated TBAPF_6_ solution in *o*F‐AB **10**. B) Change in the (*Z*)‐isomer content during electrochemical back isomerization. C) Electrochemical set‐up. With a flow rate of 1 mL min^−1^ and 1 mL inner volume of the cell, a residence time of 1 min was maintained.

In order to realize electrochemical back‐conversion, we were able to dissolve ≈5% by weight of the organic salt tetra‐*n*‐butyl ammonium hexafluorophosphate (TBAPF_6_) in the neat *o*F‐AB **10** (**Figure** **9**A. TBAPF_6_ is a salt often used for electrochemical measurements in organic solvents to increase conductivity.^[^ ^49^ ^]^ Using a simple multimeter circuit analyzer, neat *o*F‐AB **10** did not show any conductivity, which increased to a measurable quantity with TBAPF_6_ (Figure S1, Supporting Information). The observable electric conductivity results in a significant current through the liquid when applying a potential. If a potential close to the redox potential of the employed photoswitch is applied, a 1e^−^ reduction to a radical anion is generally observed.^[^ ^50^ ^]^ As the formed radical is *inter alia* located on the N═N bonds, a significant decrease in the bond order is observed.^[^ ^51^ ^]^ This results in a very low inversion barrier of (*Z*)‐ to (*E*)‐AB radical, and therefore a fast thermal isomerization.^[^ ^52^ ^]^ A subsequential electron transfer from the formed (*E*)‐isomer to a (*Z*)‐isomer, results in an electron catalytic isomerization behavior.^[^ ^48^ ^]^ This represents an alternative trigger to irradiation, acids or catalysis, which can be used to induce the heat release in MOST materials,^[^ ^53^ ^]^ and can be started either reductively or oxidatively.^[^ ^48^, ^54^, ^55^ ^]^ Azoheteroarenes with ionic side chains showed electro catalytic switching in the condensed phase, but the large isomerization barrier of these T‐shaped (*Z*)‐isomers make on demand heat release not feasible.^[^ ^56^ ^]^ We tested the electro‐catalytic isomerization of neat *o*F‐AB **10** with TBAPF_6_ *in fluxu* using a continuous flow electro‐cell. The cell had an inner volume of ≈1 mL, and with a flow rate of 1 mL min^−1^, resulted in a residence time of 1 min. Depending on the applied potential, a significant decrease in the (*Z*)‐isomer can be observed (Figure 9B). The isomerization does not take place below 2 V. At higher potentials, a nearly complete back‐isomerization is observed. These high potentials might lead to side reactions, like hydrazines or anilines by over‐reduction, although we did not observe notable degradation. We assume that the high necessary potential is due to the low intrinsic conductivity of the liquid, and the significant distance between the electrodes in the used setup. Nevertheless, complete isomerization can be obtained by this electrochemical setup. In comparison to the photochemical back reaction, which resulted in still 22% (*Z*)‐isomer after irradiation, this improvement allows harvesting of the total stored thermal energy.

**Figure 10:**
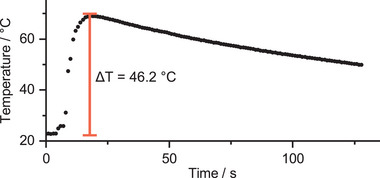
Macroscopic heat release of 1 mL *o*F‐AB **10** after the addition of a catalytic amount of LDA measured by an in situ thermometer.

To demonstrate the applicability of *o*F‐AB **10**, we isomerized ≈1 mL of the compound using a green LED. Complete heat release was achieved by addition of a catalytic amount of lithium diisopropylamide (LDA) and the resulting isomerization to the ground state was monitored using an infrared (IR) camera and an in situ thermometer. LDA is known to reduce AB,^[^ ^57^ ^]^ and we hypothesized that the intermediate radical would induce the heat release in a catalytic fashion.^[^ ^48^ ^]^ In this way, LDA can act as a heat release trigger similar to the electrochemical method, with the advantage that complete isomerization can be induced without the need for specialized equipment or setup. Upon addition of the reducing agent (at 3 s), a drastic increase in the internal temperature was observed, and the maximal temperature of 69 °C was reached within 16 s (**Figure** **10**; Video S1, Supporting Information). A similar result was obtained by the addition of a catalytic amount of hydrochloric acid (Figure S21 and Video S2, Supporting Information). A second addition of reducing agent did not result in a temperature increase over 30 °C, which indicates that the heat originated mostly in the relaxation of the (*Z*)‐isomer, and not by a reaction enthalpy.

### 2.6 Liquid MOST as Solvent

**Figure 11:**
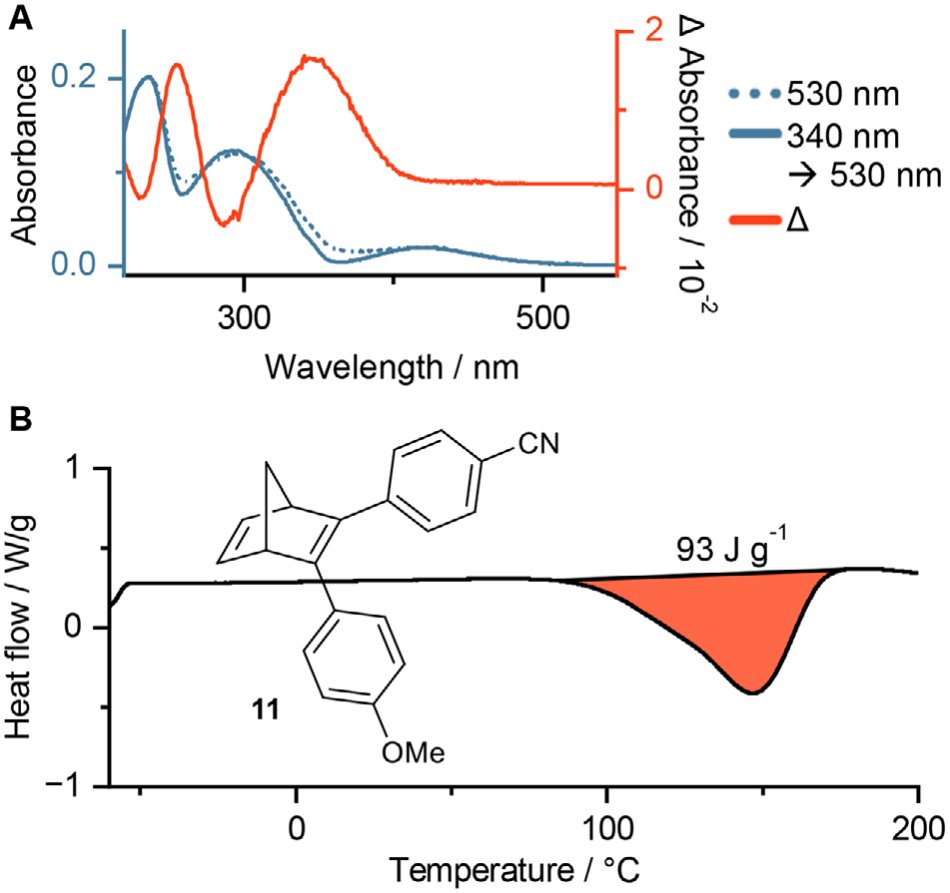
A) Absorption of the AB **10** and NBD **11** mixture in ACN. Irradiation at 530 nm switches only AB **10** while 340 nm switches both compounds. The differential spectra between the PSS at 530 nm before and after a 340 nm irradiation corresponds to the absorption spectra of NBD **11**,^[^ ^59^ ^]^ and supports the dissolution and the ability to switch of the mixture. B) DSC curve of AB **10** and NBD **11** mixtures with 58% (*Z*)‐**10**, and 18% QC‐**11**. Higher PSS were not obtainable due to decomposition of compound **11** during irradiation with 340 nm. A small peak shoulder shows the heat release of the NBD **11** (Figure S9, Supporting Information). The mixture contained NBD **11** decomposition products.

One additional scope of application for the low viscous *o*F‐AB **10** is to use it as a MOST‐active solvent. Besides additional additives, which can be added to *o*F‐AB **10** to further enhance its properties, it can be used as a solvent for higher‐energy density photoswitches. One of the disadvantages of AB for MOST storages is the relatively low stored energy in the nitrogen–nitrogen bond isomerization, in comparison to other MOST materials, that rely on bond formation, like azaborines or NBDs.^[^ ^6^, ^58^ ^]^ We used the solvent properties of AB **10** to dissolve a high energy density NBD within the AB **10**. Thereby, we can harvest the higher energy density of NBD moieties, while still handling a pumpable and easy to irradiate, low viscous liquid. In other words, we can substitute the generally employed death‐weight organic solvents with a MOST‐active solvent that does not influence the obtainable energy density negatively. As a proof‐of‐concept, we tested this new strategy by preparing an asymmetric di‐aryl NBD, as they show absorption maxima between the π–π^*^ and the n‐π^*^ absorption bands of *o*F‐AB **10**.^[^ ^59^ ^]^ The 4‐methoxyphenyl‐4′‐benzonitrile NBD **11** derivative was chosen, as it showed a good compromise between maximum absorption and half live.^[^ ^59^ ^]^ We dissolved 10% of NBD **11** in the *o*F‐AB **10**, without any observable change in the viscosity. The UV/Vis spectrum is overshadowed by the absorption of the AB moiety, which has a rather high extinction coefficient. To demonstrate that the NBD **10** is dissolved and switching in the *o*F‐AB, the mixture was irradiated first with 530 nm to switch only the AB, then with 340 nm to switch AB and NBD, followed by 530 nm again. A differential spectrum of both green light PSS spectra shows the expected absorption of NBD **11** which correlated to the measured spectra in organic solvents (**Figure** **11**A).^[^ ^59^ ^]^

Exact quantification can be achieved using ^1^H‐NMR, and PSS measurement using HPLC. A diluted sample of this mixture shows a PSS_340 nm_ of ≈95% (*Z*)‐AB **10** and 92% QC‐**11** (Figure S12, Supporting Information). For DSC measurements, we irradiated the sample neat with a 340 nm LED in a vial under stirring. Here we observed only slow isomerization of both photoswitches, and were not able to achieve a PSS state before the NBD **11** showed degradation during HPLC measurements (Figure S13, Supporting Information). This highlights the importance of *vide supra* described *in fluxu* irradiation using a flow setup. Nevertheless, we performed DSC measurements of our mixture containing only 58% (*Z*)‐*o*F‐AB **10**, and 18% QC of compound **11**. On the first glance one large exothermic peak is observed (Figure 11B). Upon closer examination, a small peak shoulder at the lower temperature region can be observed, which can be assigned to the isomerization of compound **11**. This shoulder is more pronounced in the first derivative of the DSC curve (Figure S9, Supporting Information). The smaller obtained heat release can be explained by the inefficient isomerization using 340 nm, and by degradation products which do not contribute to the energy release.

Nevertheless, we herein employed a high energy density MOST material in our MOST‐active *o*F‐AB **10** as a solvent. This adds another possibility to enhance the energy density of MOST materials, alongside approaches such as the introduction of a phase change,^[^ ^60^ ^]^ stabilization of the (*E*)‐isomer by, e.g., London dispersion interactions^[^ ^19^ ^]^ and destabilization of the (*Z*)‐isomer by templating on crowded nanocarbon.^[^ ^61^ ^]^

## 3 Conclusion

Herein, we introduced 2,6‐difluoro AB **10** as a liquid storage material for MOST systems. The compound shows isomerization upon green light irradiation, and has an energy density of 218 kJ kg^−1^ This corresponds to an energy content of 137 kJ kg^−1^ at the PSS [(*Z*)‐content 63%] with 530 nm. The storage capacity of 218 kJ kg^−1^ is considerably higher than comparable liquid AB materials, due to low molecular mass of the employed compound. The molecular energy density of 47 kJ mol^−1^ is lower than for the alkoxy AB **4**,^[^ ^22^ ^]^ but higher than arylazopyrazole **5**.^[^ ^30^ ^]^ This is in agreement with the expected stabilization of the (*Z*)‐isomer of *o*F‐AB **10** by *ortho*‐fluorination, which is less effective than the stabilization of the T‐shaped metastable isomer of AB **5**. Additionally, the obtained storage energy is similar to previously calculate energy difference between the (*E*)‐ and (*Z*)‐isomers of 45 kj mol^−1^ for AB **10**.^[^ ^28^ ^]^ Pristine AB shows a storage energy of 48 kj mol^−1^, which is higher than for AB **10**. This again can be explained by the lack of (*Z*)‐isomer stabilization in unsubstituted AB. In comparison to tetra‐*ortho* substituted ABs reported by the Group of Han, the disubstituted *o*F‐AB **10** shows higher per *mole* density. One reason would be the better stabilization of the (*E*)‐isomer by the symmetrical tetra substituted.^[^ ^16^ ^]^ The phase‐change or liquid *ortho* substituted compounds also rely on the introduction of long alkyl chain, diminishing their per *mass* energy density even further, although the thereby obtained additional latent heat storage exceeds the storage potential of *o*F‐AB **10**.

The most important feature is the low viscosity of the photoswitch of only ≈10 mPas, which allows a very efficient isomerization, handling and storing in the neat state. Especially the isomerization *in fluxu* using a peristaltic pump, a home‐made weaved tubular reactor and a cheap commercial LED strip used for ambient light irradiation, demonstrates the ease of use of *o*F‐AB **10**. To showcase the large‐scale applicability of this switch, an efficient *in fluxu* preparation via Baeyer–Mills reaction of difluoro nitrosobenzene **8** and aniline **9** in a continuous flow setup was established. After destillative purification, a throughput of 3.7 g h^−1^ pure AB **10** can be achieved. To further increase its applicability as an energy storage material, AB **10** was investigated as a MOST‐active solvent. We were able to dissolve ≈5 wt.% TBAPF_6_ conductive salt in neat AB **10**, which increased its electrical conductivity to a measurable quantity. This improvement resulted in an efficient electron‐catalyzed back isomerization using a flow electrochemical cell, which resulted in a complete back conversion to the (*E*)‐isomer. This is an improvement in comparison to the photochemical back isomerization. Here, PSS still contains considerable amounts of (*Z*)‐isomer [PSS_405 nm_ = 78% (*E*)‐isomer]. Additionally, we prepared the higher energy density NBD **11**, which we dissolved in the liquid AB **10** (10 wt.%) while still having a low viscous liquid. Complete isomerization of this mixture with 340 nm was not achieved, as the employed NBD **10** showed decomposition before PSS states could be reached. This could be improved by choosing a more stable NBD derivative with suitable absorption properties and a long half live. Nevertheless, as a proof‐of‐concept we demonstrated the strategy to dissolve and irradiate a MOST candidate dissolved in a neat MOST‐active solvent. This way, we can avoid the usage of additional solvents maintaining the processibility keeping a high absolute storage density.

## 4 Experimental Section

The detailed experimental processes are available in the Supporting Information.

## Conflict of Interest

The authors declare no conflict of interest.

## Supporting information



Supporting Information

Supplemental Video 1

Supplemental Video 2

## Data Availability

The data that support the findings of this study are available in the supplementary material of this article.
