# Supplementary material for: An Azobenzene‐Based Liquid Molecular Solar Thermal (MOST) Storage System–Energy Carrier and Solvent
Source: Small. 2025 Jun 2;21(31):2502938. doi: 10.1002/smll.202502938 (PMC12332809; doi:10.1002/smll.202502938)
Supplement: Supplementary file 1 — Supporting Information [file SMLL-21-2502938-s003.pdf]

NANO · MICRO
small

Supporting Information

for *Small*, DOI 10.1002/smll.202502938

An Azobenzene-Based Liquid Molecular Solar Thermal (MOST) Storage System–Energy Carrier and Solvent

*Dominic Schatz, Conrad Averdunk, Rouven Fritzius and Hermann A. Wegner**

An Azobenzene-Based Liquid Molecular Solar Thermal (MOST) Storage System – Energy Carrier and Solvent

Dominic Schatz,[a, b] Conrad Averdunk,[a, b] Rouven Fritzius[a, b] and Hermann A. Wegner*[a, b]

[a] D. Schatz, C. Averdunk, R. Fritzius, Prof. Dr. H. A. Wegner
Institute of Organic Chemistry
Justus Liebig University Giessen
Heinrich-Buff-Ring 17, 35392 Gießen (Germany)
E-mail: hermann.a.wegner@org.chemie.uni-giessen.de

[b] D. Schatz, C. Averdunk, R. Fritzius, Prof. Dr. H. A. Wegner
Center of Materials Research (ZfM/LaMa)
Justus Liebig University
Heinrich-Buff-Ring 16, 35391 Giessen (Germany)
35392 Gießen (Germany)

Table of Contents

General Information 3

Azocoupling reactions 4

2,6-Difluoro nitrosobenzene (**8**) – small scale 4

2,6-Difluoro nitrosobenzene (**8**) – large scale 4

2,6-Difluoro azobenzene (**10**) – small scale 5

2,6-Difluoro azobenzene (**10**) – large scale 5

2,6-Difluoro azobenzene (**10**) – continuous flow synthesis 6

NBD Synthesis 7

2,3-Dibromobicyclo[2.2.1]hepta-2,5-diene (**13**) 7

2,3-Dibromobicyclo[2.2.1]hepta-2,5-diene (**14**) 7

2-Chloro-3-(4-methoxyphenyl)bicyclo[2.2.1]hepta-2,5-diene (**16**) 8

4-(3-(4-Methoxyphenyl)bicyclo[2.2.1]hepta-2,5-dien-2-yl)-benzonitrile (**11**) 8

NMR spectra 9

^{1}H-NMR of *o*F-AB (**10**) in $CDCl_3$ 9

^{19}F-NMR of (*E*)- and (*Z*)-*o*F-AB (**10**) in $CDCl_3$ 10

^{19}F-NMR of *o*F-AB (**10**) saturated with $TBAPF_6$ in $CDCl_3$ 11

^{1}H-NMR of NBD (**11**) in CD_2Cl_2 12

Solubility NBD (**11**) in *o*F-AB (**10**) 13

Kinetics 14

Photostability 15

Additional DSC data 16

DSC of *o*f-AB (**10**) with a (*Z*)-content of 56% 16

First Derivative of DSC 16

HPLCs 17

PSS of *o*F-AB (**10**) at 530 nm irradiation 17

(*Z*)-content of *o*F-AB (**10**) for DSC measurement 17

PSS of the mixture *o*F-AB (**10**) and NBD (**11**) in ACN solutions at 340 nm irradiation 18

(*Z*)-content of *o*F-AB (**10**) and QC content of NBD (**11**) for DSC measurement 18

Degradation of neat NBD (**11**) solution in *o*F-AB (**10**) 19

Powder XRD 20

Flow irradiation 22

Photoreactor 22

Home made photoreactor 22

Macroscopic heat release 23

Single crystal XRD analysis 24

Azocoupling side product 25

References 30

Author Contributions 30

General Information

Chemicals were used as purchased from Sigma-Aldrich, Acros Organics, Alfa Aesar, TCI Europe and BLD Pharm. Anhydrous solvents were purchased from Acros Organics. Technical grade solvents used during workup and purification were distilled prior to use. Air and/or water-sensitive reactions were carried out under Schlenk-conditions. Solids were dried under high vacuum when necessary. Flash column chromatography and column chromatography was carried out with Silica 60 M (0.04 – 0.063 mm) or Silica 60 (0.063 – 0.2 mm) from Macherey Nagel GmbH & Co. KG, or basic Alox (0.05-0.2 mm, Brockmann I) from Acros Organics. Thin layer chromatography was performed on Polygram® SIL G/UV254 from Macherey Nagel GmbH & Co. KG. NMR spectra were measured on a Bruker Avance II 200 MHz, Avance II 400 MHz, Avance III 400 MHz HD, Avance III 600 MHz or spectrometer at room temperature (rt). Chemical shifts are reported in parts per million (ppm) relative to the solvent peak, coupling constants (*J*) are reported in Hertz (Hz). Deuterated solvents were obtained from Deutero GmbH (Kastellaun, Germany) or Euriso-Top GmbH. For all azobenzenes, the thermodynamically more stable (*E*)-isomer is reported if not noted otherwise. Some smaller peaks in the spectra might be due to the formation of (*Z*)-isomer. Flow reactions were performed using a Vapourtec E-Series System. The red/blue color in the pump schemes represents the inner tubings of the peristaltic pumps according to Vapourtec's color scheme. The reactions were performed in a tubular reactor with an inner volume of 10 mL. FEP-tubing (inner diameter 0.75 mm, purchases from Techlab) was used for other connections. The T-Mixer used for the reaction towards the azobenzene had an inner diameter of 1.0 mm Photochemical flow reactions were done in a Vapourtec photoreactor (standard powered LED with a wavelength of 525 nm and 3 W/ 405 nm and 9 W), or with a weaved reactor and LED strips. HPLC analysis was performed using a Shimadzu LCMS-2020 system equipped with a 150 × 4 mm Eurospher 100-5 C_{18} column with acetonitrile/water 80:20 as isocratic eluent (1.0 mL/min). Detection for PSS was carried out using a Shimadzu SPD-M20A diode array detector at the isosbestic points. Electrochemical back-reaction was performed in an ElectraSyn flow cell with graphite electrodes and a Keysight E36104B power supply. Differential scanning calorimetry was performed on a Netzsch DSC 200 F3 machine with an attached liquid nitrogen dewar. The DSC curves were corrected by subtracting an empty crucible run, and a baseline correction. Viscosities were measured with a MCR 92 rheometer from Anton Paar. For visualizing the temperature increase upon back-isomerization, a Seek Thermal Compact XR IR camera was used.

Azocoupling reactions

2,6-Difluoro nitrosobenzene (8) – small scale

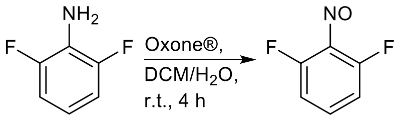

7 **8**

Adapted from a literature procedure.[1]
To a solution of 2,6-difluoroaniline (**7**, 5.5 mL, 50 mmol, 1.0 equiv.) in DCM (200 mL) was added Oxone® (61.5 g, 100 mmol, 2.00 equiv.) dissolved in H_2O (600 mL). The biphasic mixture was stirred vigorously for 4 h. Afterwards, the layers were separated and the organic phase was washed with H_2O and brine. The solvent was removed and the resulting beige solid used as is for the next steps.

Yield: 6.90 g; 96%.

2,6-Difluoro nitrosobenzene (8) – large scale

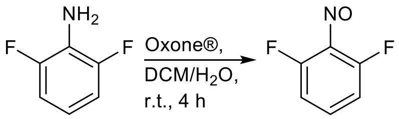

7 **8**

Adapted from a literature procedure.[1]
To an ice-cold solution of 2,6-difluoroaniline (**7**, 71.6 g, 538 mmol, 1.00 equiv.) in DCM (600 mL) was added Oxone® (661 g, 1.08 mol, 2.00 equiv.) dissolved in H_2O (1.4 L).* The biphasic mixture was stirred vigorously for 4 h. Afterwards, the layers were separated and the organic phase was washed with H_2O and brine. The solvent was removed and the resulting beige solid used as is for the next steps.

Yield: 69.9 g, 91%.

*The oxidation at these scales get hot enough to boil the DCM layer and should therefore be adequately cooled. If the reaction temperature us too high purity and yield of the nitroso compound will decrease.

2,6-Difluoro azobenzene (10) – small scale

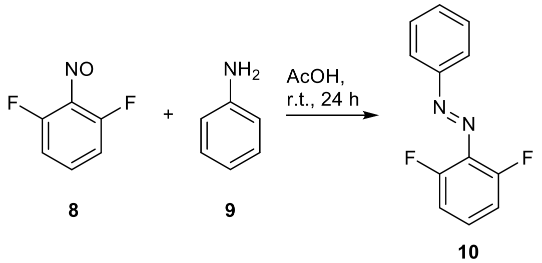

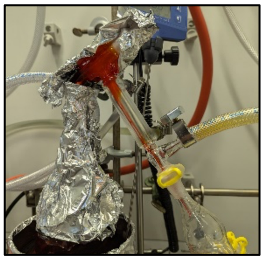

Adapted from a literature procedure.[1]
To a solution of 2,6-difluoronitrosobenzene (**8**, 6.90 g, 48.2 mmol, 1.00 equiv) in AcOH (300 mL, degassed) aniline (**9**, 4.57 mL, 50.0 mmol, 1.00 equiv.) was added. The mixture was stirred at rt for 24 h. The reaction mixture was poured over ice, NaCl was added to the aqueous phase and extracted with EtOAc (5 x 50 mL). The combined organic phases were washed with H_2O, saturated $NaHCO_3$ solution and brine. The solvent was removed and the crude mixture was purified by fractionated vacuum distillation. Pure product **10** was distilled as a red oil at 1 mbar and 140 °C heat bath temperature.

Yield: 10.4 g; 95%.

$t_{retention}$ (C_{18}-column, ACN:H_2O 8:2) = (*E*)-isomer 2.4 min, (*Z*)-isomer 1.6 min

^{1}H-NMR ($CDCl_3$, 400 MHz): δ 7.99 – 7.90 (m, 2H), 7.58 – 7.49 (m, 3H), 7.37 – 7.27 (m, 1H), 7.11 – 6.98 (m, 2H) ppm.
^{19}F-NMR ($CDCl_3$, 400 MHz): δ -121.8 ppm.

Analytical data corresponds to literature.[1]

2,6-Difluoro azobenzene (10) – large scale

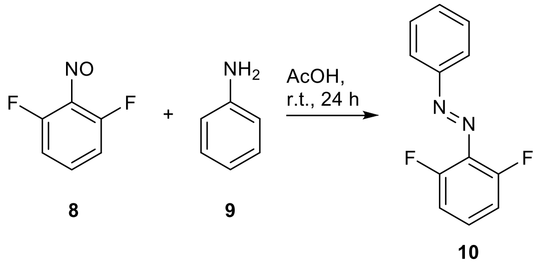

Adapted from a literature procedure.[1]
Following the above-described procedure, 2,6-difluoronitrosobenzene (**8**) was prepared from 2,6-difluoroaniline **7** (26.7 g, 203 mmol).
The crude nitrosobenzene **8** was suspended in AcOH (100 mL, degassed) and aniline (**9**, 18.5 mL, 203 mmol, 1.00 equiv.) was added. The reaction mixture was stirred at rt for one day. The solvent was removed and the crude product **10** was distilled as a red oil at 1 mbar and 140 °C heat bath temperature.

Yield: 37.6 g; 85% over two steps from anlinine **7**.

Analytical data corresponds to literature.[1]

2,6-Difluoro azobenzene (10) – continuous flow synthesis

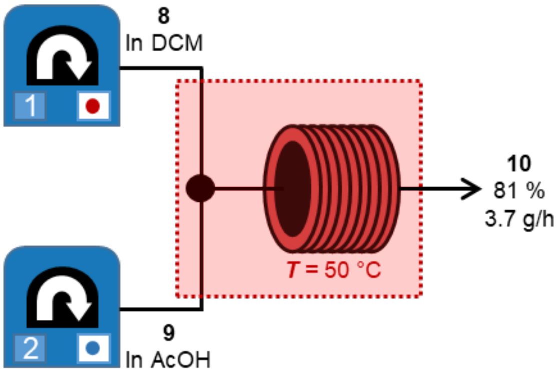

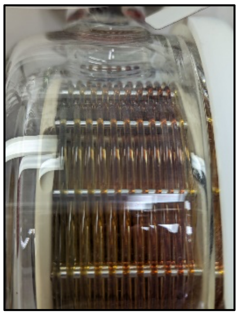

Flow-setup adapted from a previously published setup.[2]
A solution of 2,6-difluoronitrosobenzene (**2**, 20.0 g, 140 mmol, 1.00 equiv.) was dissolved in DCM (400 mL) and degassed. Aniline (13.4 mL, 147 mmol, 1.05 equiv.) was dissolved in AcOH (400 mL) and degassed. Both solutions were pumped individually at a flow rate of 1 mL/min with two V3-pumps using the Vapourtec E-Series. The streams were mixed in a T-mixer with an inner diameter of 1.0 mm and fed into a tubular reactor (10 mL) at 40 °C under pressure using a BPR (75 psi). After passing through the reactor, the reaction mixture was passed into a separatory funnel containing cyclohexane and water. The reaction was completed after 400 min, and the phases were separated. The organic phase was reduced by evaporation under pressure and the resulting crude mixture was distilled as a red oil at 1 mbar and 140 °C heat bath temperature.

Yield: 24.8 g; 81%.

NBD Synthesis

2,3-Dibromobicyclo[2.2.1]hepta-2,5-diene (13)

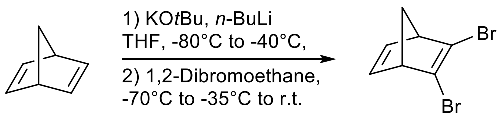

12 **13**

Adapted from a literature procedure.[3]
In a flame-dried 3-neck flask, KO*t*Bu (3.0 g, 26 mmol, 0.52 eq.) was suspended in THF (dry, 45 mL) and cooled to -78 °C. NBD (**12**, 5.1 mL, 50 mmol, 1.0 equiv.) was added. A solution of *n*-BuLi (1.6 M in hexane, 16 mL, 26 mmol, 0.52 eq.) was added dropwise over 15 min. The yellow solution was stirred at -40 °C for 1.5 h, then cooled to -78 °C and D was added in two portions. The reaction mixture was warmed to -40 °C and stirred for 30 min, then warmed to rt and stirred overnight. The mixture was quenched with addition of aquous saturated NH_4Cl solution. After addition of H_2O, the reaction mixture was extracted with Et_2O, and the combined organic phases were washed with H_2O and brine. The solvent was removed and the crude was purified by vacuum distillation at 100 °C and 10 mbar to yield the product **13** as a colorless oil.

Yield: 860 mg, 7%.

^{1}H-NMR ($CDCl_3$, 200 MHz): δ 6.89 (t, *J* = 1.6 Hz, 2H), 3.62 (p, *J* = 1.8 Hz, 2H), 2.45 (dt, *J* = 6.3, 1.6 Hz, 1H), 2.18 (dt, *J* = 6.3, 1.8 Hz, 1H) ppm.

Analytical data corresponds to literature.[3]

2,3-Dibromobicyclo[2.2.1]hepta-2,5-diene (14)

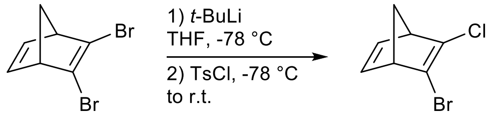

13 **14**

Adapted from a literature procedure.[3]
In a flame-dried Schlenk tube, NBD **13** (0.85 g, 3.4 mmol, 1.0 equiv.) was dissolved in THF (dry, 20 mL) and cooled to -78 °C. A solution of *t*-BuLi (1.7 M in hexane, 4.2 mL, 7.1 mmol, 2.1 equiv.) was added dropwise and the mixture was stirred at -78 °C for 15 min. Then, TsCl (0.71 g, 3.7 mmol, 1.1 equiv.) was added at once, stirred at -78 °C for 1 h followed by rt for 1 h. The mixture was quenched with H_2O, and the phase was extracted with Et_2O. Combined organic phases were washed with H_2O and brine, and the solvent removed. The crude product was rotated onto celite®, and purified by medium pressure chromatography (100% cyclohexane) to give the product **14** as a slightly yellow oil.[3]

Yield: 491 mg, 70%.

^{1}H-NMR ($CDCl_3$, 200 MHz): δ 6.94 – 6.85 (m, 2H), 3.66 – 3.57 (m, 1H), 3.56 – 3.47 (m, 1H), 2.48 – 2.38 (m, 1H), 2.23 – 2.14 (m, 1H) ppm.

Analytical data corresponds to literature.[3]

2-Chloro-3-(4-methoxyphenyl)bicyclo[2.2.1]hepta-2,5-diene (16)

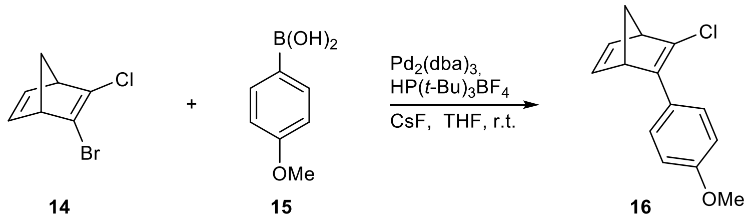

14 **15** **16**

Adapted from a literature procedure.[4]
In a flame-dried Schlenk tube, NBD **14** (0.49 g, 2.4 mmol, 1.0 equiv.), anisole **15** (0.35 g, 2.3 mmol, 0.95 equiv.), $Pd_2(dba)_3$ (68 mg, 72 µmol, 0.030 equiv.), $HP(\textit{t}\text{-Bu})_3BF_4$ (50 mg, 0.17 mmol, 0.071 equiv.) and CsF (1.3 g, 8.4 mmol, 3.5 equiv.) were added. The solids were vacuum/vented three times. Then, THF (dry, 5 mL) was added and the mixture was stirred at rt for 24 h. The mixture was filtered (eluding with DCM), and the solvent was removed. The crude product was rotated onto celite, and purified by medium pressure chromatography (100% cyclohexane) to give the product **16** as a slightly yellow solid.

Yield: 150 mg, 27%.

^{1}H-NMR ($CDCl_3$, 200 MHz): δ 7.61 – 7.50 (m, 2H), 6.98 – 6.84 (m, 4H), 3.97 – 3.88 (m, 1H), 3.82 (s, 3H), 3.57 – 3.50 (m, 1H), 2.38 – 2.28 (m, 1H), 2.16 – 2.07 (m, 1H) ppm.

Analytical data corresponds to literature.[4]

4-(3-(4-Methoxyphenyl)bicyclo[2.2.1]hepta-2,5-dien-2-yl)-benzonitrile (11)

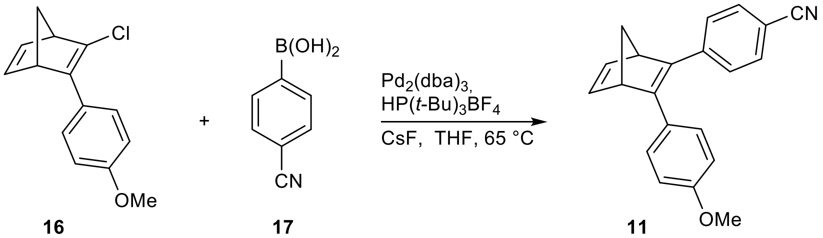

16 **17** **11**

Adapted from a literature procedure.[4]
In a flame-dried Schlenk tube, NBD **16** (0.15 g, 0.65 mmol, 1.0 equiv.), benzonitrile **17** (0.11 g, 0.71 mmol, 1.1 equiv.), $Pd_2(dba)_3$ (18 mg, 19 µmol, 0.029 equiv.), $HP(\textit{t}\text{-Bu})_3BF_4$ (14 mg, 45 µmol, 0.069 equiv.) and CsF (0.34 g, 2.3 mmol, 3.5 equiv.) were added. The solids were vacuum/vented three times, then THF (dry, 5 mL) was added and the mixture was stirred at r.t. for 24 h. The mixture was filtered (eluding with DCM), and the solvent was removed. The crude product was rotated onto celite®, and purified by medium pressure chromatography (100% cyclohexane) to give the product **11** as a yellow oil that solidifies to a waxy yellow solid.

Yield: 46 mg, 24%.

$t_{retention}$ (C_{18}-column, ACN:H_2O 8:2) = NBD 2.8 min, QC 3.1 min

^{1}H-NMR (CD_2Cl_2, 400 MHz): δ 7.53 – 7.47 (m, 2H), 7.34 – 7.29 (m, 2H), 7.15 – 7.09 (m, 2H), 7.01 – 6.94 (m, 2H), 6.83 – 6.76 (m, 2H), 3.87 (m, 2H), 3.78 (s, 3H), 2.33 (m, 1H), 2.08 (m, 1H) ppm.

Analytical data corresponds to literature.[4]

NMR spectra

^{1}H-NMR of *o*F-AB (10) in $CDCl_3$

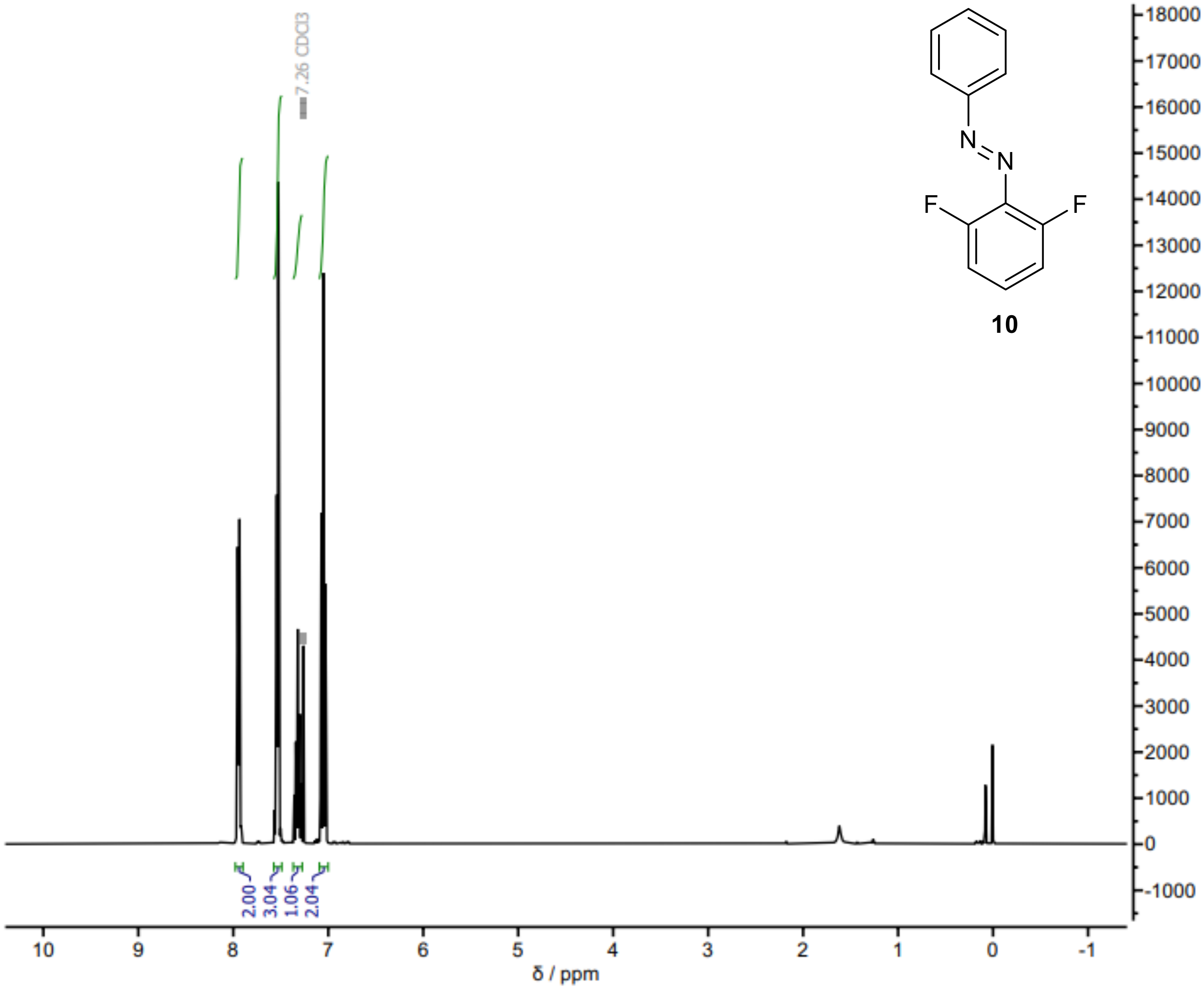

^{19}F-NMR of (*E*)- and (*Z*)-*o*F-AB (10) in $CDCl_3$

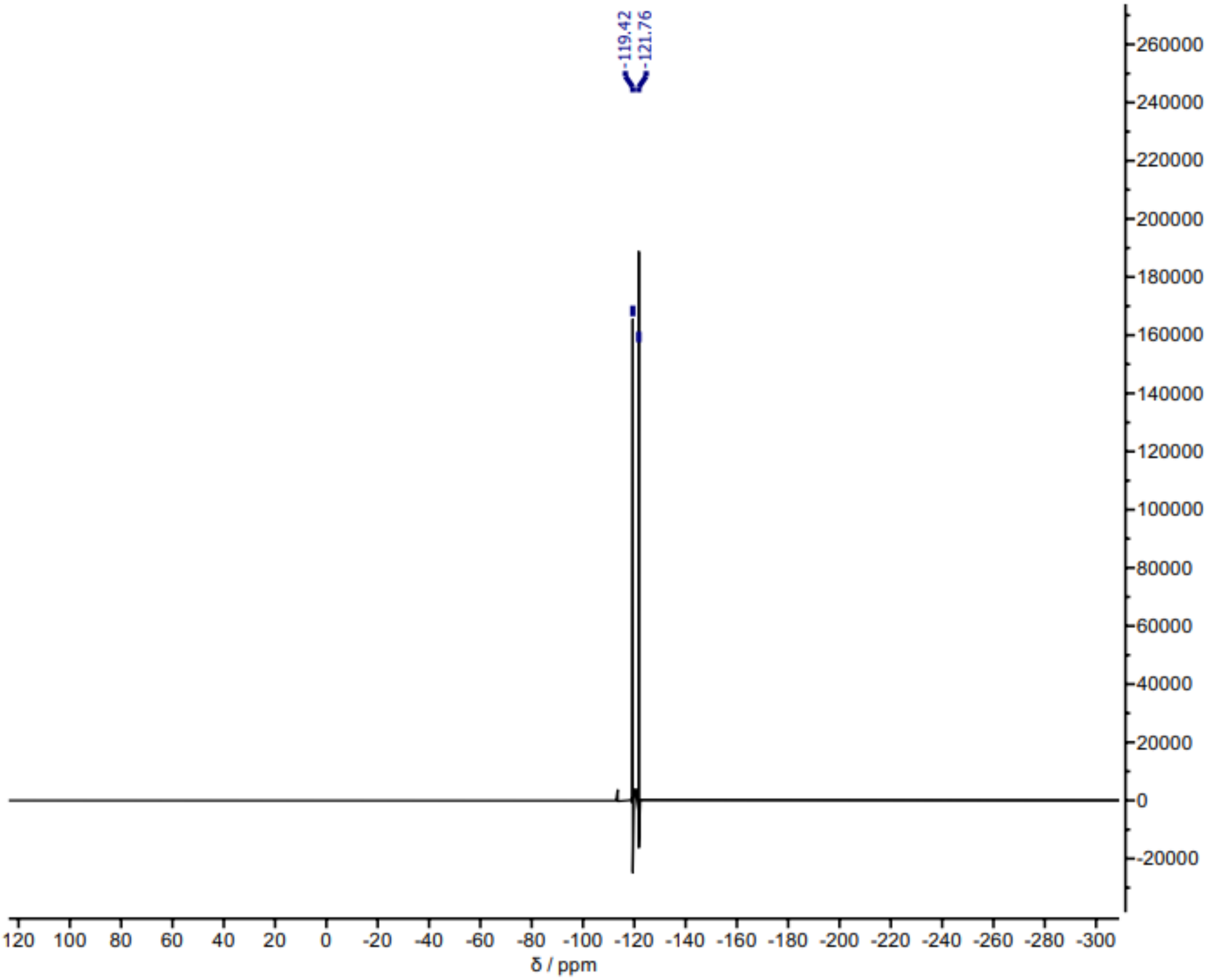

^{19}F-NMR of *o*F-AB (10) saturated with TBAPF$_6$ in CDCl$_3$

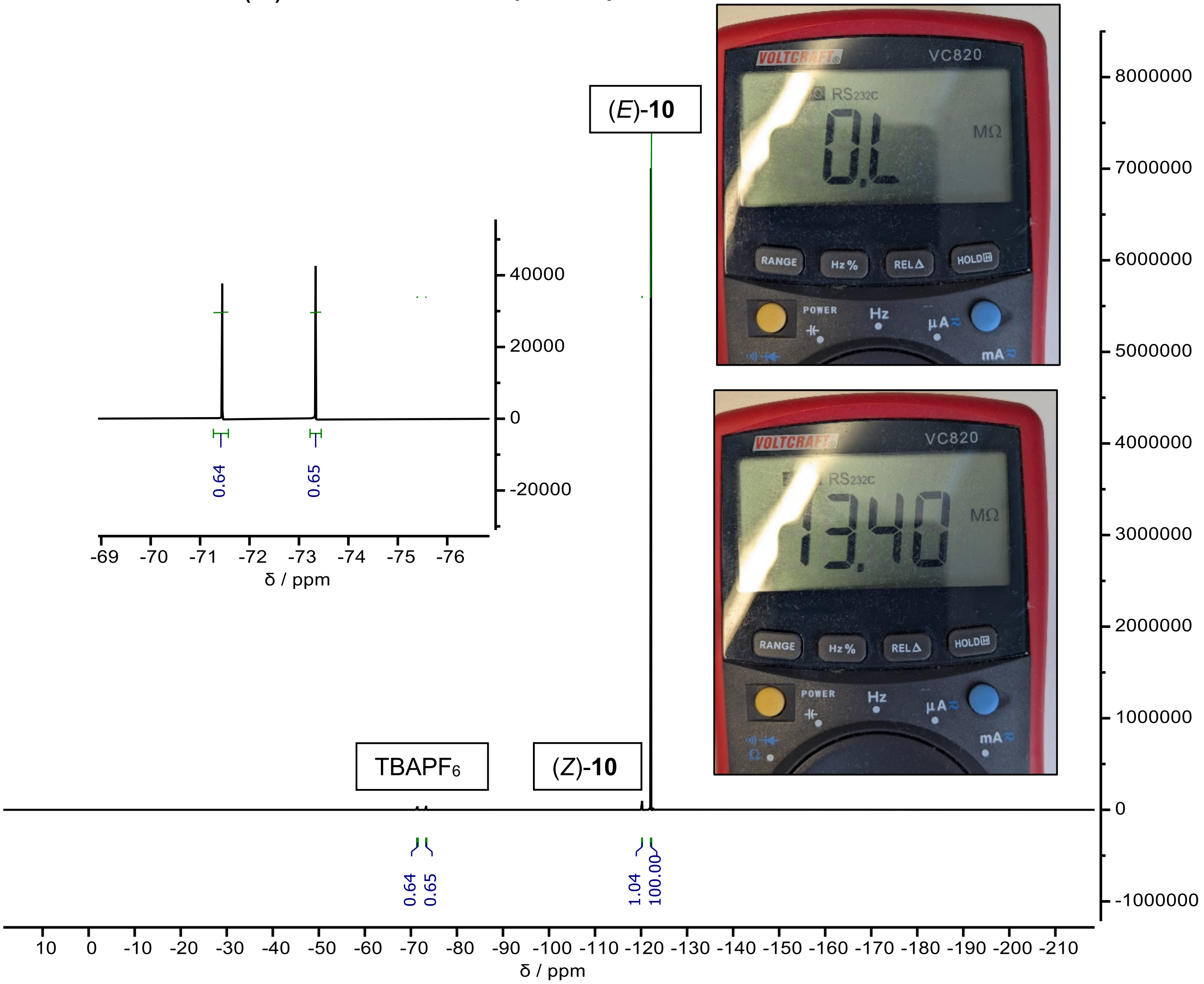

Figure S1. ^{19}F-NMR of saturated TBAPF$_6$ in *o*F-AB (**10**) measured in CDCl$_3$. By addition of the electrolyte, the non-conductive neat AB **10** (top picture) becomes a conductive liquid (bottom picture).

^{1}H-NMR of NBD (11) in CD_2Cl_2

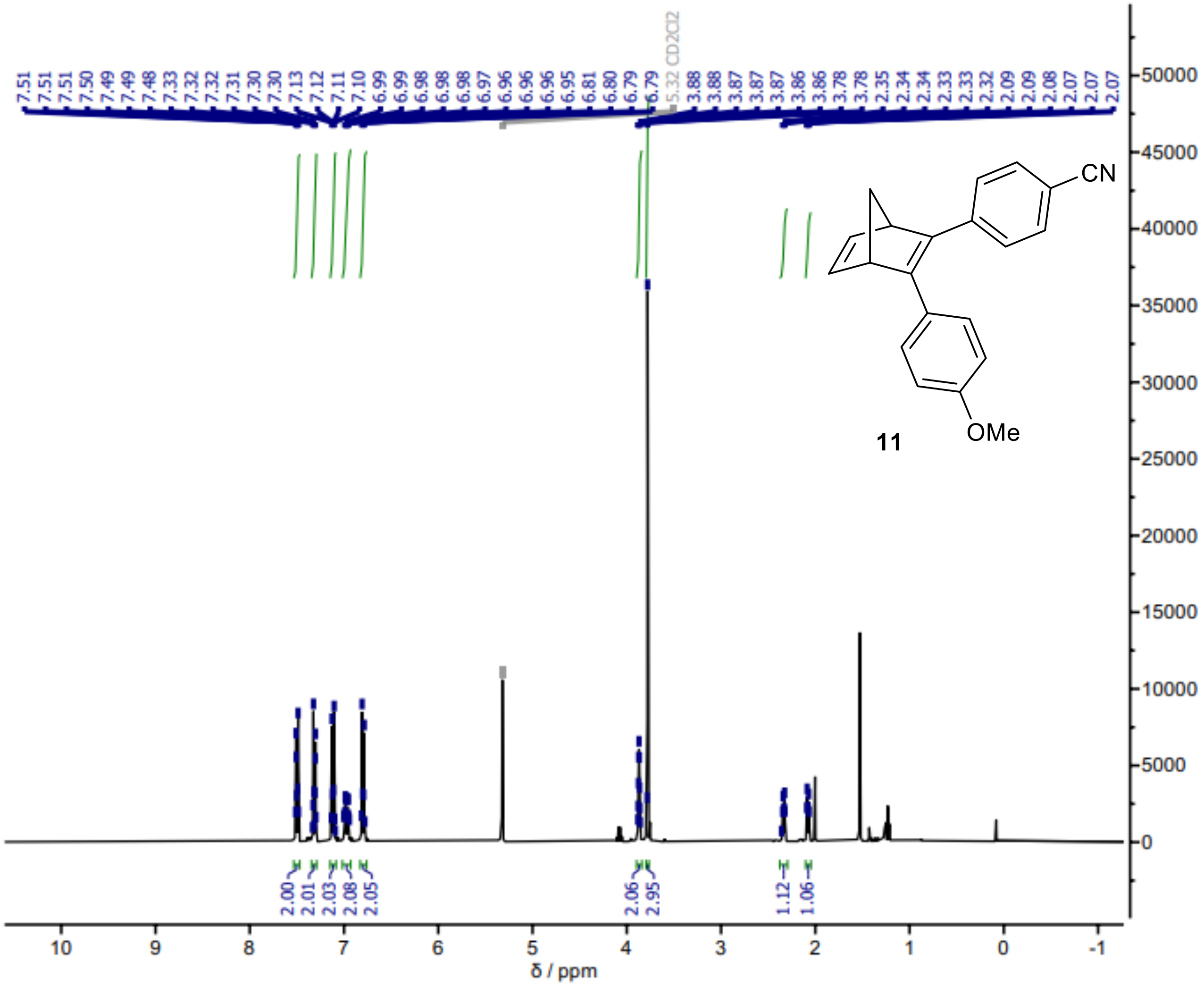

Solubility NBD (11) in *o*F-AB (10)

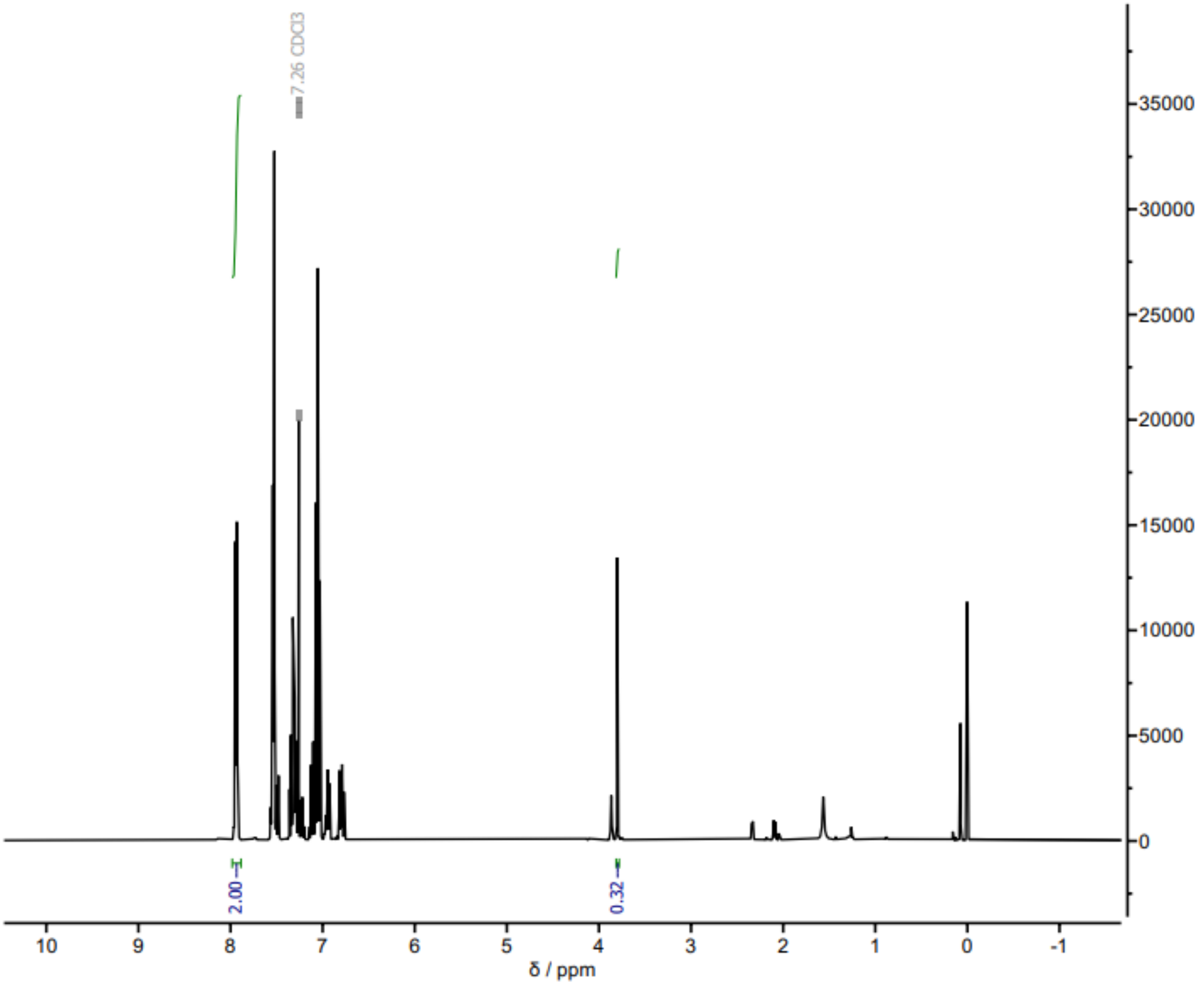

Kinetics

A neat sample of $PSS_{530\ nm}$ *o*F-AB **10** in a vial was placed in an oil bath preheated to 60 °C, 66 °C, and 70 °C and heated in the dark. An aliquot was taken at a given time, diluted in ACN, and PSS was measured *via* HPLC at the isosbestic point of the isomerization (257 nm). The (*Z*)- to (*E*) ratio was plotted over time. Measurements were conducted as a singlet. Two outliers for the 60 °C measurement were omitted. Following a previously published protocol,[5] calculation of the half-lives and kinetic constants was conducted with Origin 9.1.0G (64-bit) by the OriginLab Corporation. The measuring points were fitted with the following ExpDec1 function of the software.

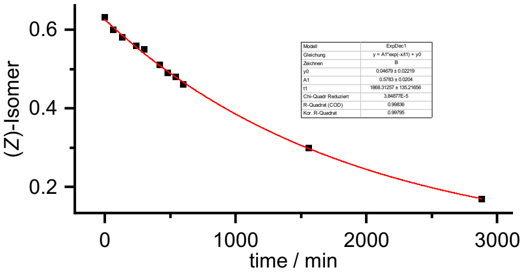

Figure S2. Fitted (*Z*)-content of neat *o*F-AB **10** at 60 °C over time.

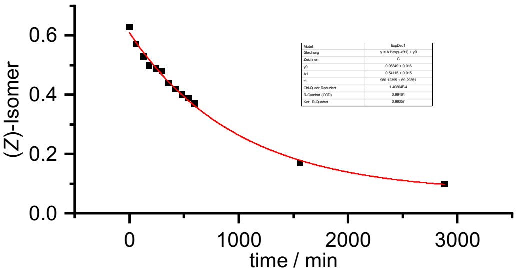

Figure S3. Fitted (*Z*)-content of neat *o*F-AB **10** at 66 °C over time.

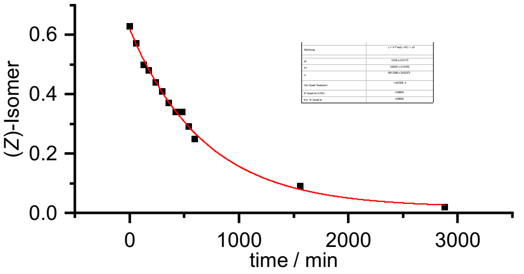

Figure S4. Fitted (*Z*)-content of neat *o*F-AB **10** at 70 °C over time.

	60 °C	66 °C	70 °C
k / min^{-1}	5.3 x 10^{-4}	1.2 x 10^{-3}	1.5 x 10^{-3}
$t_{1/2}$ / min	1295	679.4	472.4
$t_{1/2}$ / h	22	11	7.9

From these values, an Eyring-Polanyi plot was constructed to determine $\Delta H^{\neq}$ as 93.6 kJ/mol, and $\Delta S^{\neq}$, as – 7.3 J mol^{-1} K^{-1} respectively. Following the Gibbs-Helmholtz equation, this yields a $\Delta G^{\neq}$ at room temperature (25 °C) of 101.8 kJ mol^{-1}, a half-life of approximately 53 days.

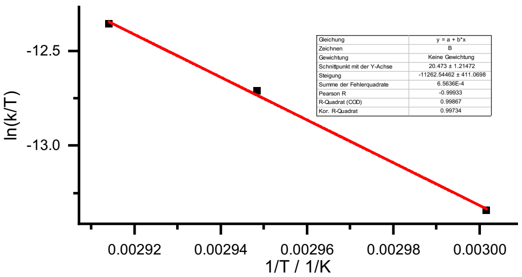

Figure S5. Eyring-Polanyi plot of *o*F-AB **10**.

Photostability

Cyclability of *o*F-AB **10** was tested in a 5 x 10^{-5} M solution in ACN. The samples were irradiated until PSS was ensured (530 nm and 340 nm for 8 min, 405 nm for 2 min) and the UV/Vis absorbance was measured. The absorbance at the maxima of 311 nm was plotted against the number of irradiation. The total irradiation times were 256 min for 530 nm and 340 nm, and 64 min for 405 nm. For green light cycling 100% maximal absorption, and for UV irradiation 99% maximal absorption was achieved after the stability tests. Absorption retention was quantified by comparing the PSS absorptions at cycle 0 and at cycle 30.

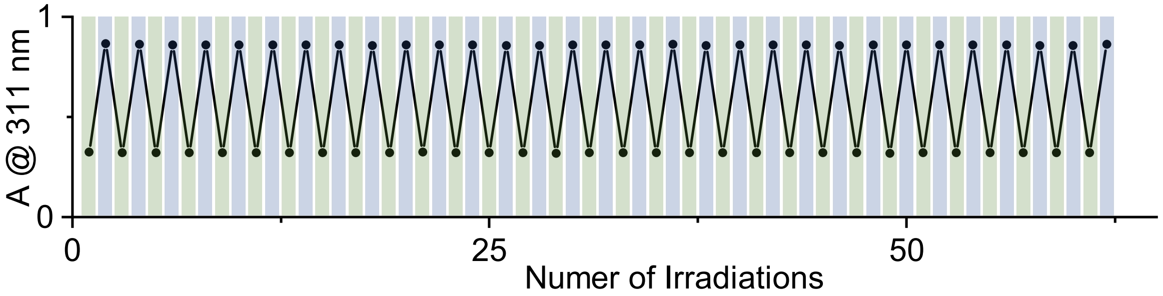

Figure S6. Absorption at 311 nm of a 5 x 10^{-5} M solution of AB **10** in ACN during the cycling. Green bars indicate 530 nm, blue bars 405 nm irradiation.

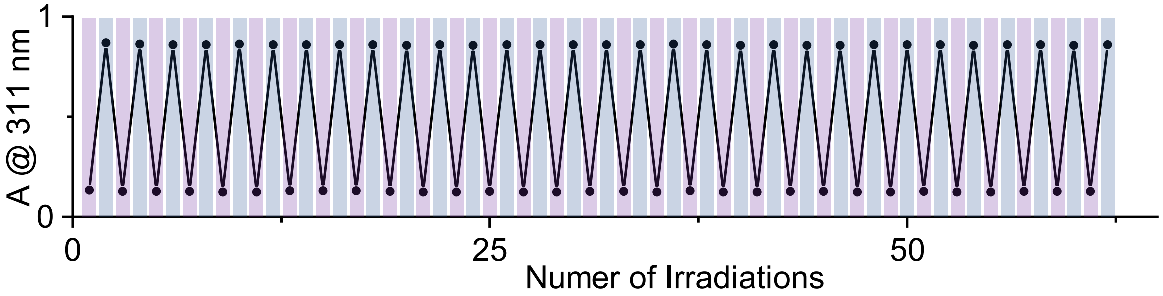

Figure S7. Absorption at 311 nm of a 5 x 10^{-5} M solution of AB **10** in ACN during the cycling. Purple bars indicate 340 nm, blue bars 405 nm irradiation.

Additional DSC data

DSC of *of*-AB (10) with a (*Z*)-content of 56%

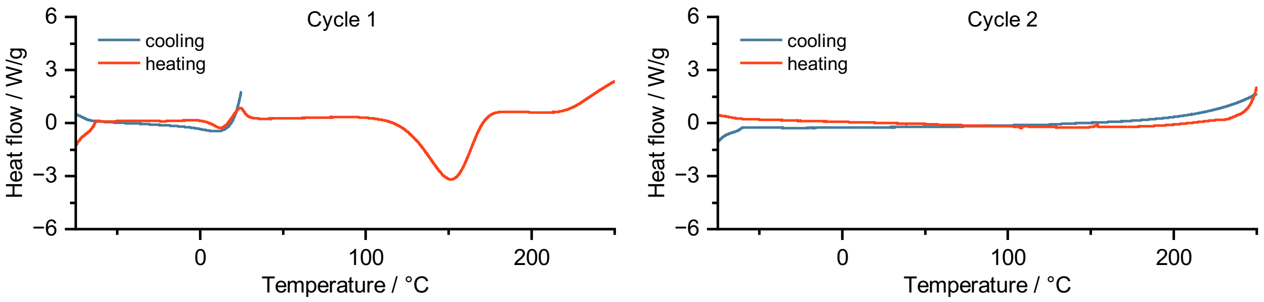

Figure S8. DSC curves of *o*F-AB (**10**) with 56% (*Z*)-content after green light irradiation. No exothermic peak was observed during the second heating cycle, indicating complete heat release.
Cycle 1: 25 °C → -75 °C → 250 °C, Cycle 2: 250 °C → -75 °C → 250 °C

First Derivative of DSC

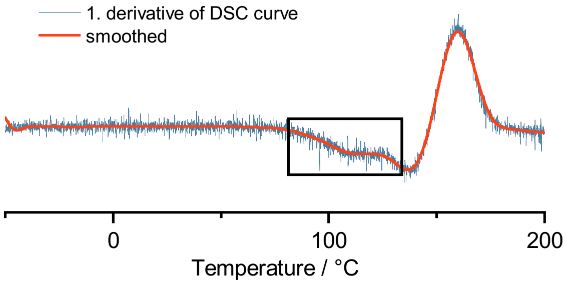

Figure S9. The first derivative and a smoothed first derivative of NBD **11** in *o*F-AB **10** mixtures after 340 nm irradiation to illustrate the shoulder in the measured DSC curve originating from the QC to NBD reaction.

HPLCs

PSS of *o*F-AB (10) at 530 nm irradiation

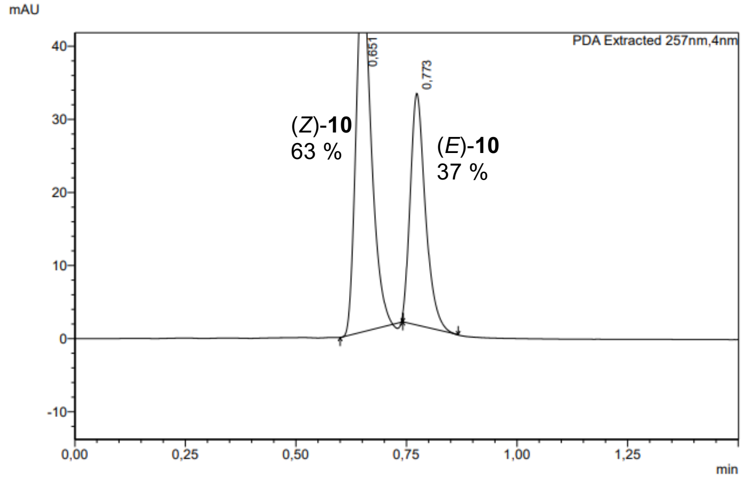

Figure S10. HPLC measurement of the PSS achievable after green light irradiation using the LED strip reactor and neat AB **10**. Integrated at 257 mm (C_{18} column, 96:4 ACN:H_2O).

(*Z*)-content of *o*F-AB (10) for DSC measurement

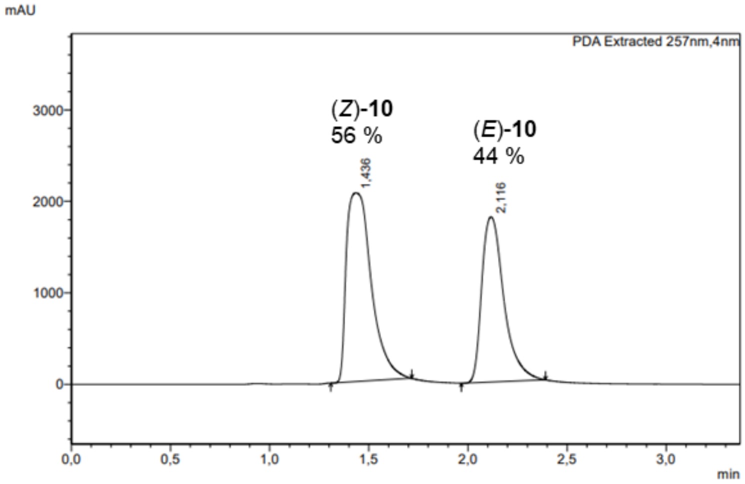

Figure S11. HPLC measurement of a sample of neat AB **10** after green light irradiation used for DSC measurements integrated at 257 nm (C_{18} column, 8:2 ACN:H_2O).

PSS of the mixture *o*F-AB (10) and NBD (11) in ACN solutions at 340 nm irradiation

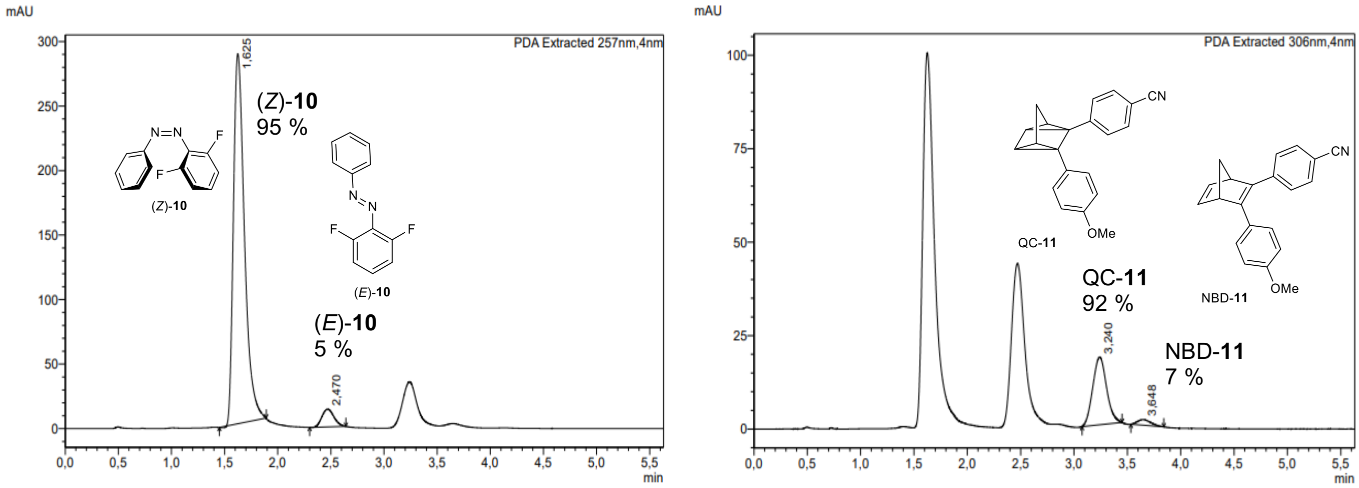

Figure S12. HPLC measurements of NBD **11** dissolved in AB **10**, diluting the mixture in ACN and irradiated with 340 nm to the PSS. AB **10** was integrated at 257 nm, and NBD **11** at 306 nm. No decomposition was observed in the chromatogram (C_{18} column, 8:2 ACN:H_2O).

(*Z*)-content of *o*F-AB (10) and QC content of NBD (11) for DSC measurement

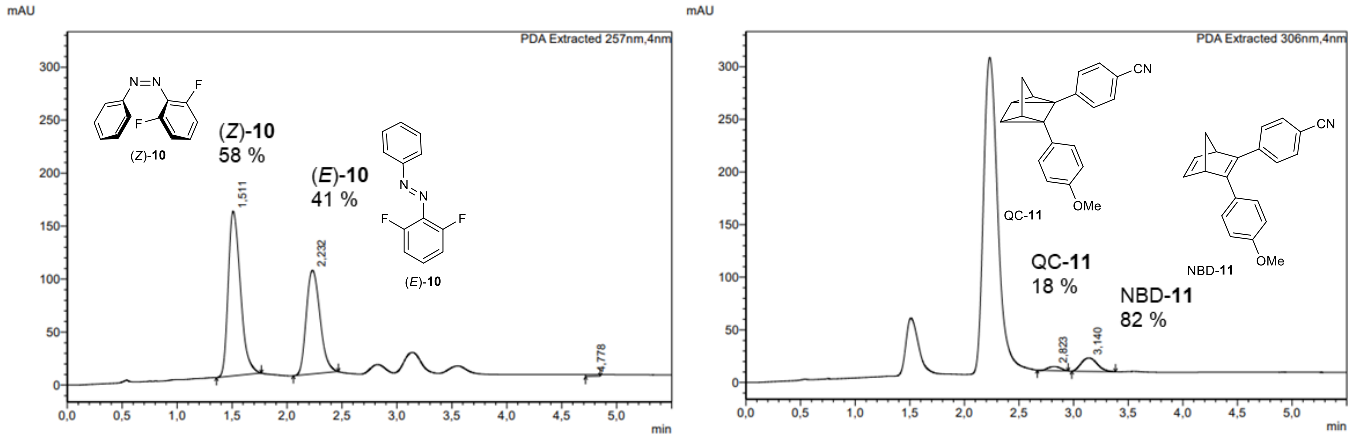

Figure S13. HPLC measurements of NBD **11** dissolved in AB **10**, irradiated with 340 nm. AB **10** was integrated at 257 nm, and NBD **11** at 306 nm. As decomposition was observed in the chromatogram, irradiation was stopped and the mixture used for DSC measurements (C_{18} column, 8:2 ACN:H_2O).

Degradation of neat NBD (11) solution in *o*F-AB (10)

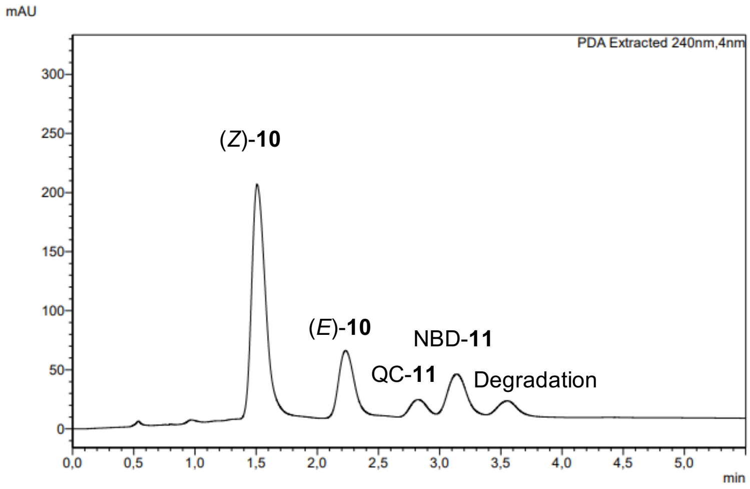

Figure S14. HPLC measurements of NBD **11** dissolved in AB **10**, irradiated with 340 nm. A new peak at 3.5 min appeared during irradiation that does not correspond to any isomer and was therefore a decomposition product. Chromatogram was extracted at 240 nm (C_{18} column, 8:2 ACN:H_2O).

Powder XRD

Temperature dependent powder XRD measurements were conducted with a STOE Stadi P diffractometer equipped with a with a focusing Ge(111) monochromator and a MYTHEN 1 K strip detector (angular range 12.5° in 2θ) in a Debye-Scherrer (transmission) geometry using Cu-Kα X-ray radiation. The sample was prepared in a quartz capillary. A Cryostream (Oxford) with a continuous-flow cold finger cooling with liquid nitrogen was used for low cooling.

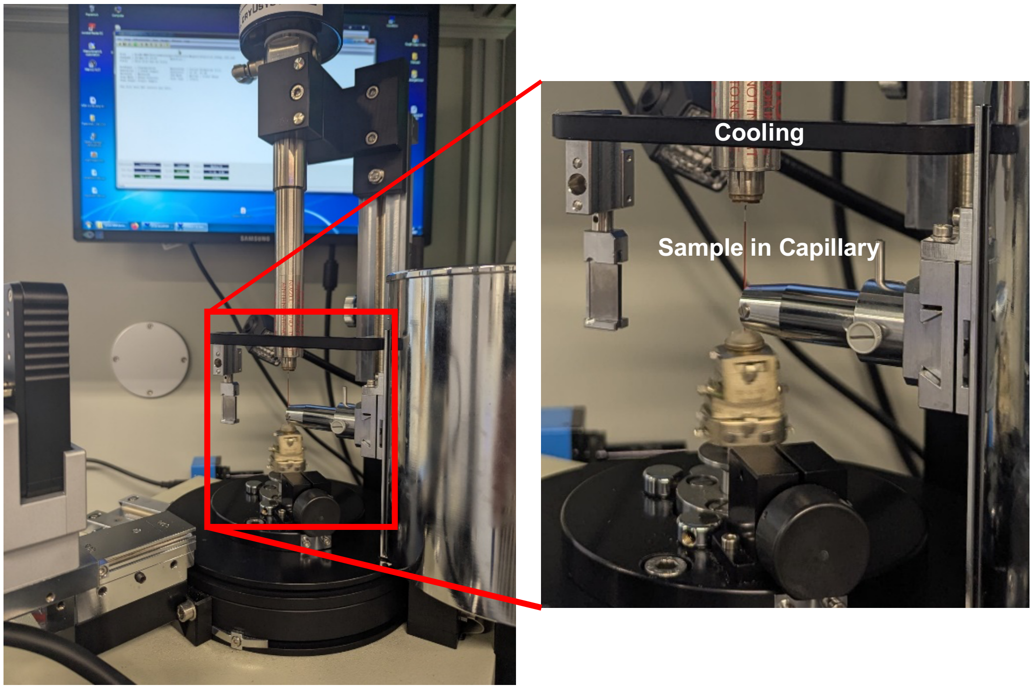

Figure S15. Set-up of the power diffractometer with home-made cooling device.

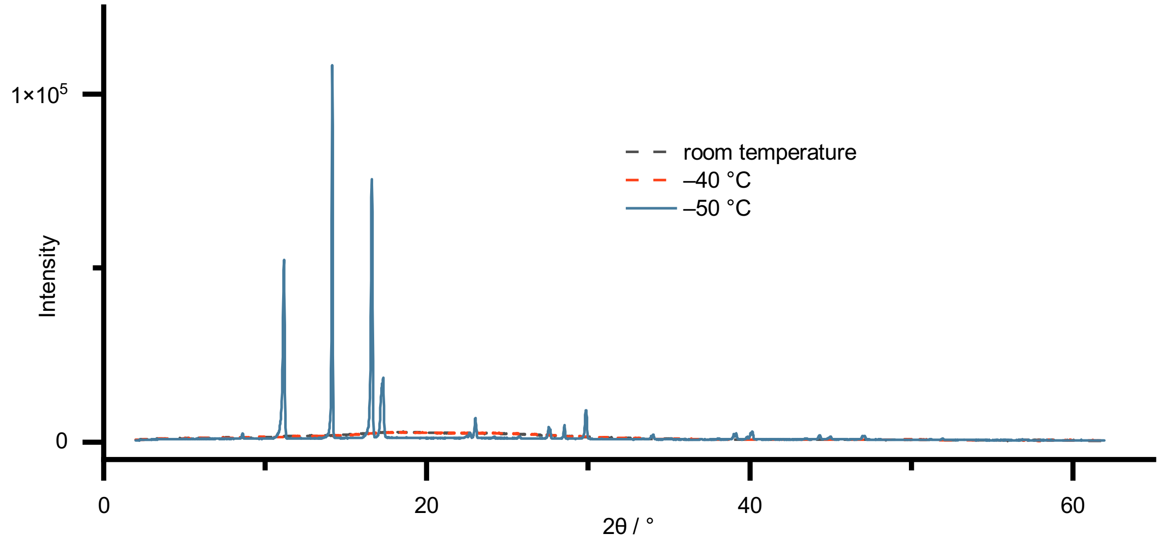

Figure S16. PXRD measurements of (*E*)-isomer of *o*F-AB **10** at room temperature, –40 °C, and –50 °C. Liquid to solid crystallization occurs between –40 °C and –50 °C.

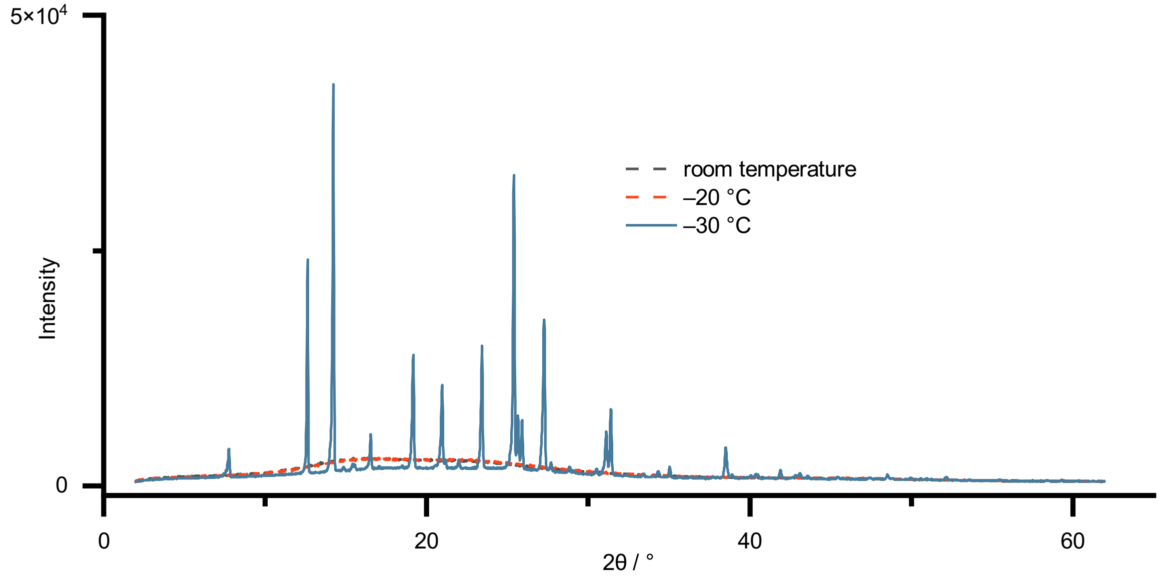

Figure S17. PXRD measurements of *o*F-AB **10** at $PSS_{530\ nm}$ at room temperature, –20 °C, and –30 °C. Liquid to solid crystallization occurs between –20 °C and –30 °C.

Flow irradiation

Photoreactor

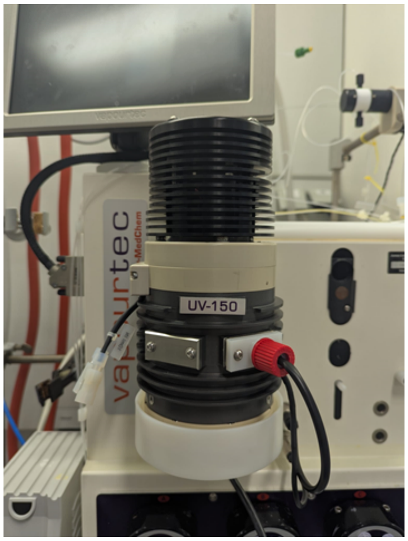

Figure S18. The UV-150 photochemical reactor from Vapurtec combined with a 405 nm LED and a 525 nm LED with 60 W input power each was used in combination with a V3 Vapourtec pump.

Home made photoreactor

FEP-tubing with an inner diameter of 0.75 mm was used to construct a 10 mL weaved tubular reactor. The reactor was weaved around a metal scaffold with minimizing overlaying tubes to increase the surface area. A LED strip normally used in ambient light applications with 20 W total power, 100 cm length and 140 LEDs was glued on top of a water-cooled trap.[6] The LED strip was placed inside the weaved reactor, which was placed inside a Dewar due to the reflective layer. The top was closed off with aluminum foil, and the reactor was connected to a SF-10 Vapourtec pump.

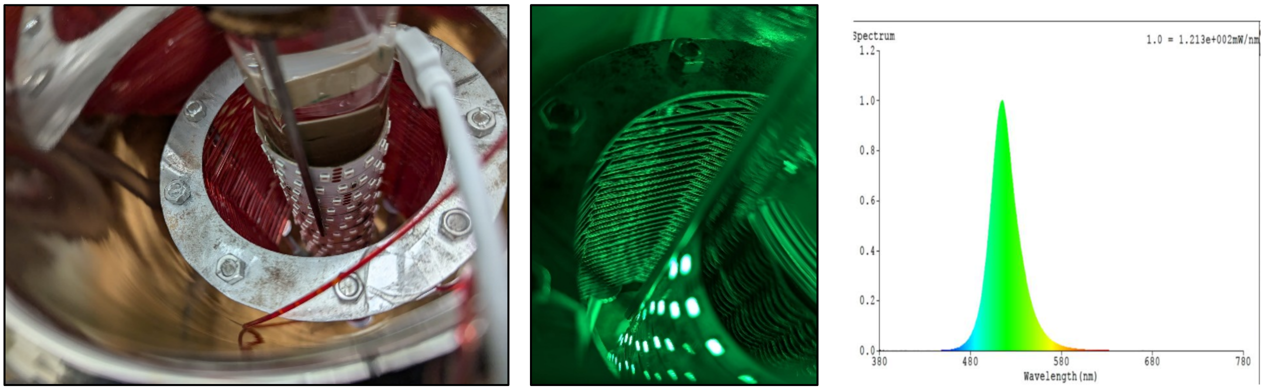

Figure S19. Homemade weaved 10 mL tubular reactor, with green light emitting LED strip inside. Emission of the LED strip is depicted on the right.[6]

Macroscopic heat release

To demonstrate the heat release qualitatively, we triggered the heat release of 1 mL $PSS_{530\,nm}$ compound using two different trigger. On one hand, the trigger was induced by addition of approx. 10% HCl dissolved in dioxane. This trigger is based on the protonation of the diazene unit to yield the azonium ion of *o*F-AB **10** that reduces the thermal half-live drastically. The second trigger was based on the addition of 10% chemical reducant lithium diisopropylamide, which reduces the AB to a radical anion with a decrease in N=N bond order and a lower isomerization barrier. These triggers were chosen as they can be used to activate the heat release easily without the need of an elaborate setup. Visualization of the heat release was followed by an IR camera. As the IR camera measured the outside temperature of the glass vial, an in situ thermometer was used to measure the temperature change more precisely. IR videos are attached to the supporting information and the heat release using acid can be found in the main manuscript. Second addition of acid did not result in any temperature increase.

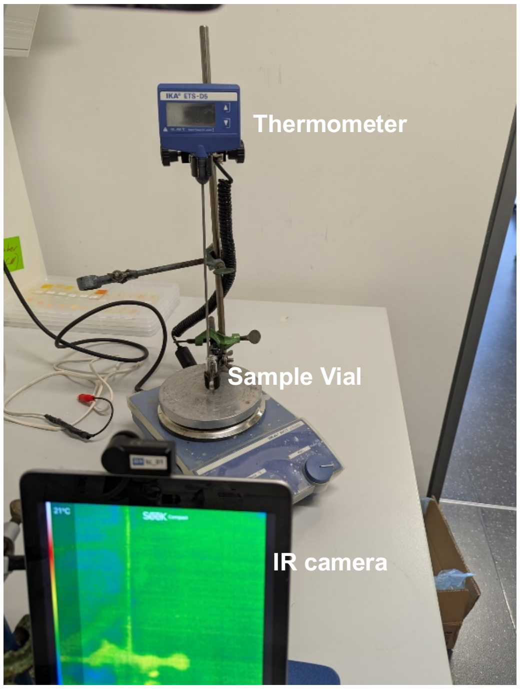

Figure S20. Setup of the triggered heat release using LDA and acid.

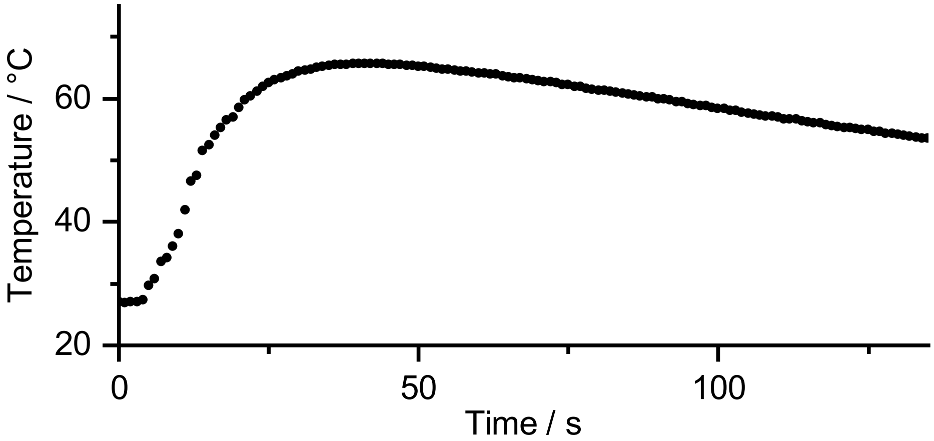

Figure S21. Macroscopic heat release after the addition of HCl.

Single crystal XRD analysis

Suitable single crystals for X-ray structure determination were selected and transferred in protective perfluoropolyether oil on a microscope slide. The selected and mounted crystals were transferred to the cold gas steam on the diffractometer. The diffraction data were obtained at 100 K on a Bruker D8 three circle diffractometer, equipped with a PHOTON 100 CMOS detector and a IµS microfocus sources with Quazar mirror optics (Mo-Kα radiation, λ = 0.71073 Å). The data obtained were integrated with SAINT and a semi-empirical absorption correction from equivalents with SADABS-2016/2 was applied.[7] The structures were solved by direct methods using SHELXT-2018/2.[8] Structure refinement was done using SHELXT-2018/3.[9] All non-hydrogen atoms were refined anisotropically and C-H hydrogen atoms were positioned at geometrically calculated positions and refined using a riding model. The isotropic displacement psarameters of all hydrogen atoms were fixed to 1.2x or 1.5x (CH_3 hydrogens) the U_{eq} value of the atoms they are linked to.

Azocoupling side product

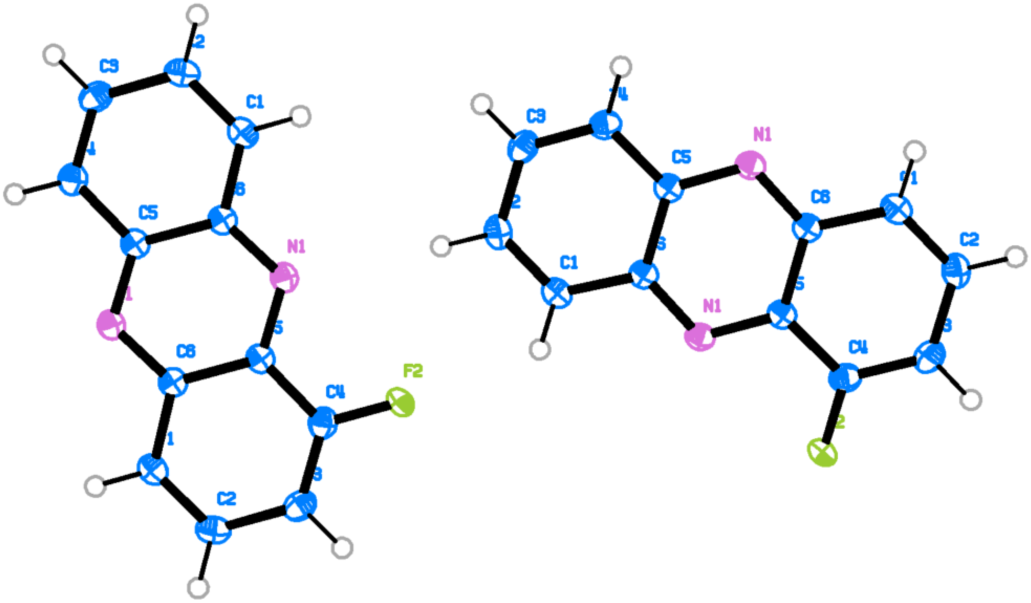

Figure S22. ORTEP structure of the side product from the azo coupling reaction with ellipsoids at 50% probability. Disordered structures are omitted for clarity.

Crystal data and structure refinement for CCDC: 2425004.

Identification code	2425004	
Empirical formula	C12 H7 F N2	
Formula weight	198.20	
Temperature	100(2) K	
Wavelength	0.71073 Å	
Crystal system	Monoclinic	
Space group	$P2_1/c$	
Unit cell dimensions	a = 3.7378(5) Å	α= 90°.
	b = 13.0087(19) Å	β= 101.186(6)°.
	c = 9.2374(13) Å	γ = 90°.
Volume	440.63(11) $Å^3$	
Z	2	
Density (calculated)	1.494 Mg/m^3	
Absorption coefficient	0.106 mm^{-1}	
F(000)	204	
Crystal size	0.737 x 0.164 x 0.145 mm^3	
Theta range for data collection	2.739 to 27.101°.	
Index ranges	-4<=h<=4, -16<=k<=16, -11<=l<=11	
Reflections collected	18970	
Independent reflections	971 [R(int) = 0.0534]	
Completeness to theta = 25.242°	99.5 %	
Absorption correction	Semi-empirical from equivalents	
Max. and min. transmission	0.745686 and 0.525914	
Refinement method	Full-matrix least-squares on F^2	
Data / restraints / parameters	971 / 0 / 80	
Goodness-of-fit on F^2	1.082	
Final R indices [I>2sigma(I)]	R1 = 0.0430, wR2 = 0.1246	
R indices (all data)	R1 = 0.0508, wR2 = 0.1361	
Extinction coefficient	n/a	
Largest diff. peak and hole	0.222 and -0.275 e.$Å^{-3}$	

Atomic coordinates (x 10^4) and equivalent isotropic displacement parameters ($Å^2$x 10^3) for 2425004. U(eq) is defined as one third of the trace of the orthogonalized U^{ij} tensor.

	x	y	z	U(eq)
N(1)	6341(3)	4048(1)	5648(1)	19(1)
C(1)	7013(3)	6830(1)	6461(1)	22(1)
F(1)	5716(5)	7708(1)	5856(2)	26(1)
C(2)	9270(3)	6819(1)	7800(1)	23(1)
C(3)	10587(3)	5861(1)	8441(1)	23(1)
C(4)	9631(3)	4956(1)	7735(1)	22(1)
F(2)	11080(40)	4117(9)	8261(15)	26(1)
C(5)	7263(3)	4948(1)	6329(1)	18(1)
C(6)	4093(3)	4092(1)	4330(1)	18(1)

Bond lengths [Å] and angles [°] for 2425004.

N(1)-C(6)	1.3398(16)
N(1)-C(5)	1.3422(15)
C(1)-F(1)	1.3219(18)
C(1)-C(2)	1.3551(17)
C(1)-C(6)#1	1.4233(15)
C(1)-H(1)	0.9500
C(2)-C(3)	1.4250(16)
C(2)-H(2)	0.9500
C(3)-C(4)	1.3594(16)
C(3)-H(3)	0.9500
C(4)-F(2)	1.274(13)
C(4)-C(5)	1.4235(17)
C(4)-H(4)	0.9500
C(4)-H(4X)	0.9500
C(5)-C(6)#1	1.4373(15)
C(6)-N(1)-C(5)	116.57(9)
F(1)-C(1)-C(2)	120.52(11)
F(1)-C(1)-C(6)#1	117.70(12)
C(2)-C(1)-C(6)#1	121.78(10)
C(2)-C(1)-H(1)	119.1
C(6)#1-C(1)-H(1)	119.1
C(1)-C(2)-C(3)	119.51(10)
C(1)-C(2)-H(2)	120.2
C(3)-C(2)-H(2)	120.2
C(4)-C(3)-C(2)	121.33(11)
C(4)-C(3)-H(3)	119.3
C(2)-C(3)-H(3)	119.3
F(2)-C(4)-C(3)	120.5(6)
F(2)-C(4)-C(5)	119.0(6)
C(3)-C(4)-C(5)	120.21(10)
C(3)-C(4)-H(4)	119.9
C(5)-C(4)-H(4)	119.9
C(3)-C(4)-H(4X)	119.9
C(5)-C(4)-H(4X)	119.9
N(1)-C(5)-C(4)	119.46(10)
N(1)-C(5)-C(6)#1	121.48(12)
C(4)-C(5)-C(6)#1	119.06(10)
N(1)-C(6)-C(1)#1	119.94(10)
N(1)-C(6)-C(5)#1	121.94(10)
C(1)#1-C(6)-C(5)#1	118.11(12)

Symmetry transformations used to generate equivalent atoms:

#1 -x+1,-y+1,-z+1

Anisotropic displacement parameters (Å^{2}x 10^3) for DK25015_a. The anisotropic displacement factor exponent takes the form: $-2\pi^2$[h^2 $a^{*2}U^{11}$ + ... + 2 h k a* b* U^{12}]

	U^{11}	U^{22}	U^{33}	U^{23}	U^{13}	U^{12}
N(1)	20(1)	19(1)	20(1)	2(1)	6(1)	1(1)
C(1)	23(1)	18(1)	25(1)	0(1)	7(1)	0(1)
F(1)	37(1)	14(1)	25(1)	1(1)	0(1)	-1(1)
C(2)	25(1)	22(1)	24(1)	-4(1)	7(1)	-5(1)
C(3)	21(1)	29(1)	19(1)	-1(1)	4(1)	-2(1)
C(4)	22(1)	24(1)	20(1)	2(1)	6(1)	1(1)
F(2)	37(1)	14(1)	25(1)	1(1)	0(1)	-1(1)
C(5)	18(1)	19(1)	19(1)	1(1)	7(1)	0(1)
C(6)	18(1)	19(1)	19(1)	1(1)	7(1)	-1(1)

Hydrogen coordinates (x 10^4) and isotropic displacement parameters (Å^{2}x 10 ^{3}) for 2425004.

	x	y	z	U(eq)
H(1)	6144	7471	6039	26
H(1X)	4300(400)	9180(100)	4550(160)	26
H(2)	9970	7444	8307	28
H(3)	12165	5856	9381	28
H(4)	10547	4327	8183	26
H(4X)	10547	4327	8183	26

Hydrogen bonds for 2425004_a [Å and °].

D-H...A	d(D-H)	d(H...A)	d(D...A)	<(DHA)
C(2)-H(2)...N(1)#2	0.95	2.58	3.4995(15)	162.2
C(2)-H(2)...F(2^d)#2	0.95	2.60	3.140(12)	116.4
C(3)-H(3)...F(1^a)#3	0.95	2.53	3.233(2)	130.8

Symmetry transformations used to generate equivalent atoms:

#1 -x+1,-y+1,-z+1 #2 -x+2,y+1/2,-z+3/2 #3 x+1,-y+3/2,z+1/2

References

[1] C. Knie, M. Utecht, F. Zhao, H. Kulla, S. Kovalenko, A. M. Brouwer, P. Saalfrank, S. Hecht, D. Bléger, *Chem. Eur. J.* **2014**, *20*, 16492–16501.
[2] J. H. Griwatz, A. Kunz, H. A. Wegner, *Beilstein J. Org. Chem.* **2022**, *18*, 781–787.
[3] S. M. Büllmann, A. Jäschke, *Chem. Commun.* **2020**, *56*, 7124–7127.
[4] V. Gray, A. Lennartson, P. Ratanalert, K. Börjesson, K. Moth-Poulsen, *Chem. Commun.* **2014**, *50*, 5330–5332.
[5] L. Schweighauser, M. A. Strauss, S. Belotto, H. A. Wegner, *Angew. Chem. Int. Ed.* **2015**, *54*, 13436–13439.
[6] zu finden unter https://www.leds24.com/24V-LED-Streifen-gruen-140-LEDs-je-Meter-alle-5cm-teilbar-?number=X108-0020#).
[7] L. Krause, R. Herbst-Irmer, G. M. Sheldrick, D. Stalke, *J. Appl. Cryst.* **2015**, *48*, 3–10.
[8] G. M. Sheldrick, *Acta Cryst. A* **2015**, *71*, 3–8.
[9] G. M. Sheldrick, *Acta Cryst. C* **2015**, *71*, 3–8.

Author Contributions

D. S. and H. A. W. conceptualized the project and prepared the manuscript. C. A. offered significant input regarding synthesis and photophysical experiments, and R. F. offered significant scientific input regarding flow chemistry. All authors were involved in discussing the data.
